# A digital twin of the infant microbiome to predict neurodevelopmental deficits

**DOI:** 10.1126/sciadv.adj0400

**Published:** 2024-04-10

**Authors:** Nicholas Sizemore, Kaitlyn Oliphant, Ruolin Zheng, Camilia R. Martin, Erika C. Claud, Ishanu Chattopadhyay

**Affiliations:** ^1^ Department of Medicine, University of Chicago, Chicago, IL 60637, USA.; ^2^ Department of Pediatrics, University of Chicago, Chicago, IL 60637, USA.; ^3^ Division of Neonatology, Weill Cornell Medicine, New York, NY 10021, USA.; ^4^ Neonatology Research, University of Chicago, Chicago, IL 60637, USA.; ^5^ Committee on Quantitative Methods in Social, Behavioral, and Health Sciences, University of Chicago, Chicago, IL 60637, USA.; ^6^ Committee on Genetics, Genomics and Systems Biology, University of Chicago, Chicago, IL 60637, USA.; ^7^ Center for Health Statistics, University of Chicago, Chicago, IL 60637, USA.

## Abstract

Despite the recognized gut-brain axis link, natural variations in microbial profiles between patients hinder definition of normal abundance ranges, confounding the impact of dysbiosis on infant neurodevelopment. We infer a digital twin of the infant microbiome, forecasting ecosystem trajectories from a few initial observations. Using 16*S* ribosomal RNA profiles from 88 preterm infants (398 fecal samples and 32,942 abundance estimates for 91 microbial classes), the model (Q-net) predicts abundance dynamics with *R*^2^ = 0.69. Contrasting the fit to Q-nets of typical versus suboptimal development, we can reliably estimate individual deficit risk (*M*_δ_) and identify infants achieving poor future head circumference growth with ≈76% area under the receiver operator characteristic curve, 95% ± 1.8% positive predictive value at 98% specificity at 30 weeks postmenstrual age. We find that early transplantation might mitigate risk for ≈45.2% of the cohort, with potentially negative effects from incorrect supplementation. Q-nets are generative artificial intelligence models for ecosystem dynamics, with broad potential applications.

## Teaser

Generative AI discovers cross-talk among gut bacteria in preterm infants that might help reduce neurodevelopmental risk.

## INTRODUCTION

The human microbiome, a complex community hosting trillions of microorganisms such as bacteria, archaea, viruses, and various microbial eukaryotes, plays a crucial role in maintaining general health and homeostasis (*1*, *2*). Increasing evidence suggests that microbial dysbiosis contributes to the development and progression of numerous diseases (*3*), ranging from facilitating essential digestive processes (*4*) to regulating the central nervous system through the microbiota-gut-brain axis (*5*–*7*) (table S1). Despite the wealth of detailed -omics profiles available on various microbiome taxa, our comprehension of the early-life ecosystem development remains limited. While the microbiome’s role in brain development (*7*) and the significance of microbial dysbiosis in neuroinflammation and neurodevelopmental disorders have been observed [including in preterm births (*8*–*10*)], the specific mechanistic pathways operating along the gut-brain axis are yet to be fully understood (*11*).

Building upon the limited understanding of the early-life microbiome development, researchers have been exploring predictive models that can help in diagnosing and understanding the implications of dysbiosis on various health outcomes in pediatric populations. Predictive models for serious pediatric intestinal diseases, such as necrotizing enterocolitis, have been studied by analyzing the infant microbiome through standard deep learning architectures (*12*, *13*). Other research has investigated the predictive diagnosis of early childhood cognitive deficits based on observed dysbiosis, typically by comparing the microbiomes of children with and without a target disorder (*14*). However, focusing on subjects already diagnosed with cognitive deficits provides limited insights into potential early clinical interventions. Data-driven identification of the microbial ecosystem’s organizational rules presents a formidable challenge (*15*); the immense complexity of potential interactions within the microbial ecosystem makes it impractical to discover its organizational rules through experimentation alone, although some targeted experiments for determining causal relationships do exist (*16*). Moreover, the relatively high cost and time-consuming nature of microbiome profile collection result in limited dataset sizes, further complicating automated inference. Current analyses often concentrate on inferring coarse correlative associations (*17*–*19*), with limited ability to discern subtle predictive patterns and nonlinear relationships (*20*, *21*).

The rapid maturation of the infant microbiome, which occurs over days to weeks, adds to the challenge by limiting the number of time-course data points that can be realistically collected. Various approaches to the longitudinal analysis of microbiome profiles have been documented, using classical statistical methods (*22*, *23*) and dynamical systems theory, such as generalized Lotka-Volterra models (*24*) and probabilistic graphical methods like dynamic Bayesian networks (DBNs) (*25*). However, applicability to the general problem of analyzing noisy, sparse, high-dimensional microbiome data may be limited (*26*). While efforts have been made to address some of these concerns (*26*–*28*), state-of-the-art results in microbiome forecasting have been largely limited to synthetic or simulated data (*24*, *27*) and nonhuman hosts (*29*–*31*) and might necessitate simplifying assumptions that limit the complexity of inferred interactions (*24*–*29*, *31*). A comparison of some major existing methods found in the literature is provided in table S2. In addition, the common lack of an out-of-sample validation cohort, which is crucial for claiming predictive ability and generalizing beyond the training data, is problematic.

Here, we hypothesize that to delineate the impact of gut microbiome maturation trajectories on cognitive development [assessed via the well-established proxy of head circumference growth (HCG)] (*11*, *32*–*37*) in preterm infants, a more profound understanding of the underlying rules of microbial organization is essential. To achieve this goal, we developed a computational framework that learns *n*-way time-aware dependencies among ecosystem inhabitants without imposing any a priori restrictions on the nature of interactions. This framework creates a digital twin of the ecosystem at the level of microbial classes for this case study (although any taxonomic level can be modeled). In engineering design, a digital twin represents a complex system comprehensively and accurately in a digital form, enabling the simulation of perturbations; study of trajectories, aberrations, and failures; and the execution of high-fidelity simulation experiments that would otherwise be unattainable in the real world. Rather than answering a single question, a digital twin aims to mirror the entire system, distinguishing it from typically more limited standard machine learning models.

We found that our digital twin, which we refer to as the Q-net, successfully generates reliable long-term forecasts of coupled trajectories for key microbial classes in realistic patient populations. Furthermore, it unveils meaningful patterns that increase the risk of future suboptimal cognitive development. The detailed dependencies across bacterial classes that we identify provide interpretable insights unattainable through a standard predictive model alone. In contrast to existing methods, our digital twins serve more than just a forecasting function; we explicitly use them to determine patient-specific risk measures that can be assessed early enough to design targeted clinical interventions, validated out-of-sample from a second independent study site.

## RESULTS

### Data source

**Fig. 1.**
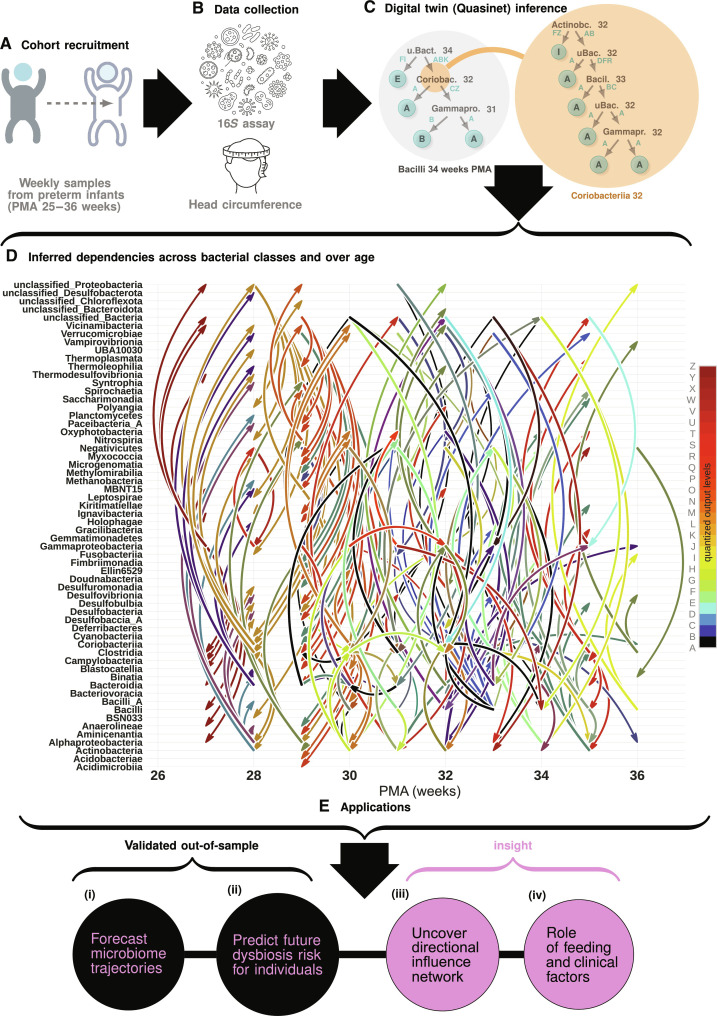
Scheme of the study. (**A** and **B**) Longitudinal fecal samples from infants born <35 weeks PMA subjected to 16*S* rRNA gene sequencing, along with infant head circumference growth (HCG) measurement. (**C**) Microbiome abundance data are quantized into 26 levels between measured maxima for each observed taxonomic class, and is used to infer a digital twin, reflecting complex emergent dependencies across the classes via learning a recursive forest of conditional inference trees (the Q-net). Component trees in the Q-net (two examples shown) are inter-dependent, where nonleaf node can recursively expand to its own tree. (**D**) Complex dependencies inferred for typically developing sub-cohort across classes and observation time points. Each inferred dependency rule probabilistically relates an a priori unspecified number of entities (>2), and together specify a generative model of ecosystem trajectory and its dynamical operation. (**E**) Applications enabled by the inferred digital twin, with out-of-sample validation and important mechanistic insights.

Our first data source comes from a cohort of 58 preterm infants born less than 35 weeks gestational age, recruited (*11*) from University of Chicago’s Comer Children’s Hospital to join the Microbiome in Neonatal Development (MIND) study, referred to as the UChicago cohort. This dataset consists of longitudinal fecal samples of microbial relative abundances obtained from the subjects via 16*S* ribosomal RNA (rRNA) gene sequencing (*38*, *39*) measured weekly from birth to term-equivalent age for each patient [24 to 36 weeks postmenstrual age (PMA), see fig. S1 for a mapping between weeks of life and PMA], as well as a variety of clinical variables such as delivery mode, feeding type, antibiotic usage, etc. (see Fig. 1 for the overall scheme of the study). Technical details of sequencing and data processing described in (*11*) are also given in the “Data sources” section and descriptions of the variables measured in the study are enumerated in table S10.

For out-of-sample validation, a second cohort of 30 preterm infants born less than 35 weeks gestational age from the MIND study were recruited at the Beth Israel Deaconess Medical Center, Boston, MA (referred to as the Boston cohort). For both cohorts, we target a clinical response of the classification of patients by level of cognitive development reflected by the proxy of head circumference growth (HCG), which is assessed here via difference in head circumference *z* score from birth to term-equivalent age (36 weeks postmenstrual age) to the Fenton growth curve (*35*, *40*).

We consider two clinical phenotypes (*11*): infants who ultimately attain appropriate (≥ −0.5 *z* score difference) versus suboptimal (< −0.5 *z* score difference) HCG (AHCG and SHCG, respectively). Overall patient characteristics are presented in Table 1. Notably, the phenotype distribution of the UChicago cohort was nearly balanced, with 28 infants classified as AHCG and 30 classified as SHCG; similarly, it was nearly balanced with respect to sex distribution: the cohort contained 30 females and 28 males. Similarly, the Boston cohort also had a relatively balanced distribution of 18 infants with AHCG and 12 infants with SHCG, and an equal male/female ratio. With respect to the observed taxonomy, across both cohorts, samples contained microbial entities from 44 unique phyla and 91 distinct classes. Table 1 contains a listing of the most abundant entities by class in separate subcohorts induced by HCG classification; notably, ≈10 entities are sufficient to capture 99% of mean relative abundance.

**Table 1.** Demographic and clinical characteristics of study subjects in MIND cohort.

Characteristic	Description			
Clinical target variable	Infant’s head circumference growth (HCG)			
Clinical cohorts (2)	Appropriate HCG versus suboptimal HCG			
Patient age range	24–36 weeks postmenstrual age (PMA)			
Frequency of data collection	≈ weekly			
Unique microbial phyla	44			
Unique microbial classes	91			
**Characteristic**	**UChicago**		**Boston**	
Number of patients	58		30	
Average fecal samples/patient	4.13		5.27	
	**UChicago**		**Boston**	
	**AHCG (*n* = 28)**	**SHCG (*n* = 30)**	**AHCG (*n* = 18)**	**SHCG (*n* = 12)**
Gestational age at birth (weeks), mean ± SD	28.32 ± 2.60	26.9 ± 2.64	29.78 ± 2.82	29.333 ± 2.99
Male, *n* (%)	13 (46.43%)	15 (50%)	9 (50%)	6 (50%)
Birth weight, g, mean ± SD	1021.96 ± 382.91	998.3 ± 423.45	1328.89 ± 394.77	1488.33 ± 632.74
Birth head circumference, cm, mean ± SD	24.875 ± 2.88	24.57 ± 3.26	27.32 ± 2.22	28.25 ± 3.27
Vaginal delivery, *n* (%)	12 (42.86%)	4 (13.33%)	6 (33.33%)	4 (33.33%)
Length of NICU stay, days, mean ± SD	77.89 ± 34.77	103.27 ± 61.21	66.13 ± 27.94	72.09 ± 33.61
Postmenstrual age at NICU discharge, weeks, mean ± SD	39.04 ± 3.80	41.13 ± 6.80	39.33 ± 2.09	39.27 ± 2.94
	**UChicago**		**Boston**	
**Microbial class (mean rel. abund.)**	**AHCG (*n* = 28)**	**SHCG (*n* = 30)**	**AHCG (*n* = 18)**	**SHCG (*n* = 12)**
Gammaproteobacteria	0.647963	0.505579	0.650564	0.519976
unclassified_Bacteria	0.076359	0.149064	0.067438	0.077718
Clostridia	0.073411	0.033252	0.027653	0.128344
Bacteroidia	0.063502	0.009749	0.018662	0.053227
Bacilli	0.046029	0.174091	0.123372	0.127355
Negativicutes	0.036066	0.044133	0.069160	0.045433
Actinobacteria	0.027100	0.031281	0.035964	0.037978
unclassified_Proteobacteria	0.009649	0.040266	0.000010	0.000083
Alphaproteobacteria	0.007616	0.003534	0.002703	0.001581
Fusobacteriia	0.006863	0.003711	0.001340	0.005913
Verrucomicrobiae	0.000532	0.000810	0.000117	0.000158
Vicinamibacteria	0.000491	0.000901	0.000547	0.000402
Nitrospiria	0.000196	0.000364	0.000159	0.000169
Oxyphotobacteria	0.000144	0.000051	0.000024	0.000074

We chose to obtain our generative model at the taxonomic level of microbial classes. To address the compositional nature of relative abundance data, we quantize all observations into a finite number of bins corresponding to quantiles of the range of relative abundance values recorded over the entire time period for the specific microbial class. Our model operates on these quantized observations; for subsequent interpretation, we map relevant quantized values back to continuous relative abundances (see the “Q-net construction from relative abundance profiles” section).

### Digital twin construction from longitudinal microbiome profiles

We construct our models using the relative abundance profiles observed in the UChicago cohort, and carry out out-of-sample validation of these models for doing long-term forecast of microbiome maturation, as well as predicting the risk of suboptimal developmental outcomes in the Boston cohort. The different digital twins produced in this study are represented in table S14. Note that the Q-net inference algorithm is not deterministic, and inferred models exhibit small variance with respect to connections and generative probabilities. Thus, to ensure robustness/validity, our results are based on an ensemble of models generated by re-fitting multiple times on the same underlying data (see the “Q-net model regeneration” section).

Relative abundances of the microbes vary under emergent constraints of competition, amensalism, cooperation, commensalism, and exploitation, and many of these dependencies are unknown or poorly understood a priori. From the observed time-stamped samples of microbiome profiles, we aim to maximally infer these complex rules (see the “Q-net construction from relative abundance profiles” section) as follows. For each patient, we treat observed relative abundances of each microbial class at a specific time point (identified by the PMA of the subject at the time of sample collection) as a distinct variable or “feature.” The Q-net, inferred from these data, consists of a set of distinct predictors, each modeling the expected probability distribution of the relative abundance of a specific variable (a microbial class at a specified time point or a class-time point pair), conditioned on the remaining variables, i.e., the rest of the microbial relative abundances recorded possibly at different time points. No locality restrictions on temporal dependence are made, and the model is free to infer dependencies present between different entities across all past time points. Thus, we may have as many estimators as the number of observed microbial classes times the number of discrete time stamps, i.e., for our dataset: 91 classes × 12 time points = 1092 (although not all classes had nonzero observations at all time points).

In each Q-net, we infer our component predictors described above as conditional inference trees (*41*, *42*), which use explicit statistical tests to ensure that each node split during the tree construction is significant at a preset level and thus limits overfitting. Each of these decision trees aims to predict the relative abundance level of a specific bacterial class at a specific time point and uses as features other bacterial classes similarly coupled with corresponding time stamps. A fragment of a Q-net is shown in Fig. 1C, where the predictor for Bacilli at 34 weeks PMA is shown on the right, which uses as features “Unclassified Bacteria at 34 weeks,” “Coriobacteriia at 32 weeks,” and “Gammaproteobacteria at 31 weeks” as predictive features; i.e., these are the variables that appear in the internal nodes of the tree. Note that the feature “Coriobacteriia at 32 weeks” has its own predictor as shown on the right, and uses as features “Actinobacteria at 32 weeks,” “Unclassified Bacteria at 32 weeks,” “Bacilli at 33 weeks,” and “Gammaproteobacteria at 32 weeks.” Thus, any nonterminal node of a component tree for an inferred Q-net can in general be “expanded” to its own tree. Owing to this recursive expansion, a complete Q-net is difficult to visualize simultaneously, but it nevertheless substantially captures the complexity of the rules shaping the ecosystem as evidenced by our out-of-sample validation. (Note that there is always imprecision with regard to the timing of these events, partly due to the possibility of ≈1- to 2-week ambiguity in postmenstrual age of the infants, and hence we consider features with time stamps 2 weeks in future to affect an entity. This “flexibility” is only allowed in the inference stage and not in testing and thus does not affect prediction results.) The set of features that a particular estimator uses is identified automatically, and the inferred ensemble of conditional inference trees maximally captures the organizational structures emergent in the observed microbiome profiles. The rich tapestry of multi-way time-aware cross-talk that we infer is depicted in Fig. 1D.

With our inferred models, we carried out five computational interrogations, namely, (i) forecasting microbiome maturation trajectories for out-of-sample subjects, with very few initial observations; (ii) out-of-sample prediction of the phenotypic fate of infants with regard to their cognitive development from their forecasted microbiome maturation trajectories to evaluate the relative risk of suboptimal development at a time point early enough for potential intervention; (iii) uncovering a directional influence network connecting the observed taxa, to determine critical ecosystem constituents that differentiate phenotype from both their relative influence on microbiome maturation trajectories and changes in network connectivity at each time point, a concept that moves beyond defining important taxa through changes in relative abundance toward their relative role in ecosystem dynamics over time; (iv) interrogating the role of key clinical factors in shaping microbiome maturation and dysbiosis over time (Fig. 1E); and (v) determining microbiome or clinical interventions that could reduce risk of SHCG on a personalized level. Reasoning with the patterns that the Q-net inferred is made possible via two key computations: (i) sampling the digital twin to impute missing data in a manner that obeys the inferred probabilistic constraints, and (ii) quantitatively assessing the dissimilarity of two distinct microbiome trajectories, via an intrinsic distance induced by the Q-net model. Here, a “microbiome trajectory” refers to time-stamped relative abundance values of all observed microbial classes, possibly with missing entries.

### Sampling the digital twin for imputation and forecasting

The inferred Q-net allows us to impute missing relative abundance values that maximally exploit the learned dependencies across taxa. Our procedure is conceptually equivalent to the well-known Gibbs sampling scheme (*43*, *44*). We can estimate the missing relative abundance value of a specific bacterial class at a given time, by drawing a sample from the conditional distribution for the corresponding variable, i.e., a class–time stamp pair, as estimated by the Q-net. As is the case for Gibbs sampling, if repeated such samples are being drawn, in the limit, we are sampling from the joint distribution of all variables, i.e., the space of all feasible microbiome trajectories. We use this imputation scheme to forecast maturation dynamics: fixing a set of initial conditions corresponding to all observed relative abundances at times *t* < 28 weeks PMA, we treat future relative abundances from 28 weeks PMA and later as unknown or missing, which we can then impute iteratively, with the new values obtained essentially being the predicted relative abundances.

### Validation and comparison of forecast performance for microbiome maturation trajectories

**Fig. 2.**
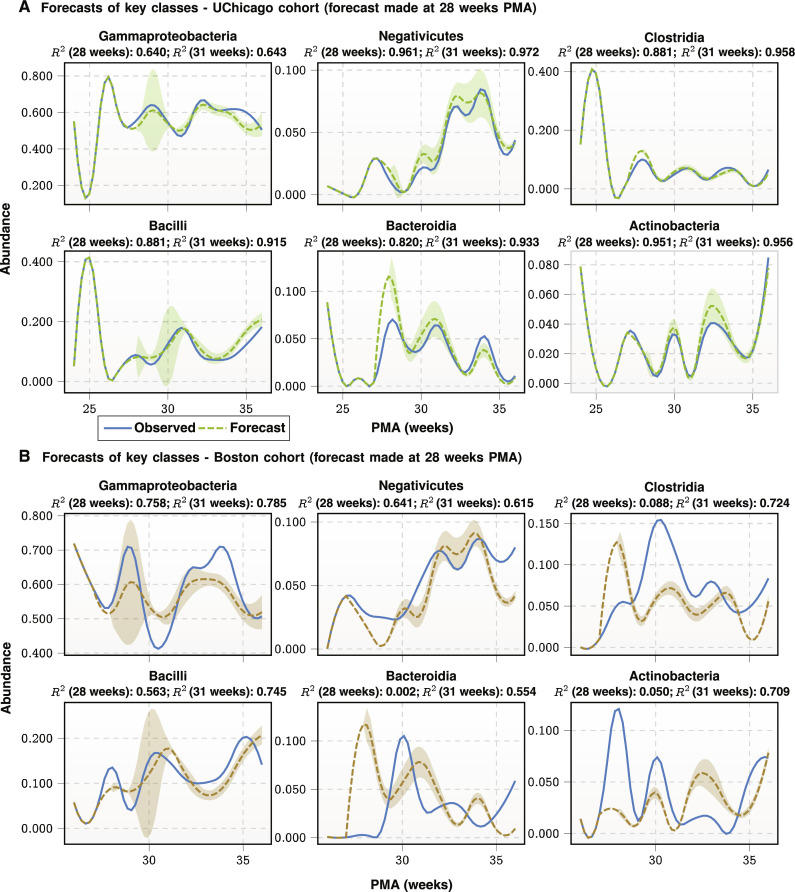
Trajectory forecasts. Population-level forecasting of mean abundance trajectories of select taxonomic classes (defined by having mean relative abundance >0.01 in the training set) from a set of observations restricted to <28 weeks PMA. (**A**) Forecasts generated from initial conditions specified by the UChicago cohort (from which the Q-net was inferred). Average *R*^2^ across these taxa is ≈0.856 at 28 weeks, and ≈0.896 at 31 weeks. (**B**) Forecasts generated using initial conditions specified by the Boston cohort (fully out-of-sample data that were not used for inference). Average *R*^2^ across these taxa is ≈0.350 (which increases to ≈0.378 when allowing for a temporal shift of 1 week) at 28 weeks, and ≈0.689 at 31 weeks. In both cases, explained variance is typically high in these important classes, suggesting that the Q-net model successfully captures the complicated dynamical trajectories.

We evaluate the quality of these predictions by assessing the coefficient of determination or the *R*-squared measure (*R*^2^) between observed mean relative abundance and model-predicted values from initial conditions specified by separate independent datasets before 28 and 31 weeks PMA. We achieve average *R*^2^ > 0.85 on forecasts of the UChicago cohort (in-sample) starting from 28 weeks PMA and average *R*^2^ = 0.89 for the 31-week PMA forecasts. For the out-of-sample Boston cohort, we achieve an average *R*^2^ = 0.69 at the 31-week PMA prediction, while the results for out-of-sample prediction at 28 weeks PMA are somewhat worse as expected (mean *R*^2^ = 0.35). The results are shown in Fig. 2, with 95% confidence bounds. It is important to note that if we allow relative shift between the predicted and observed time courses of ±1 week, then the mean *R*^2^ = 0.52 for predictions at 28 weeks (the prediction at 31 weeks does not improve with this possibility of relative shifts), indicating some degree of uncertainty in PMA estimates of the infants, especially for those who are born earlier than 30 weeks. Increased challenge of determining PMA for early preterm births has been reported before (*45*).

To place these results in context, we compute the coefficient of determination *R*^2^ obtained by DBNs, which represent the state-of-the-art framework in longitudinal forecasting of relative abundances of microbial taxa. We implemented a direct comparison (see the “Forecasting coupled microbiome maturation” section) of our Q-net–based approach to DBNs, exploring various levels of DBN model complexity. These results are shown in fig. S2 and table S4, which depict the forecasting performance of DBNs of increasing depth from two-stage to six-stage architectures on both the UChicago cohort (in-sample) and Boston cohort (out-of-sample). Forecasts are made at 28 and 31 weeks PMA for all models; we found that all of the DBNs evaluated attained *R*^2^ less than half of the corresponding Q-net–based approach. This substantial reduction in *R*^2^ is demonstrative of a concurrent substantial reduction in the ability of DBNs to model these data in comparison to the Q-net approach.

### Quantifying future risk *M*_δ_ of anomalous deviation in microbiome maturation

In our study, we recognize that the infant microbiome maturation trajectory is influenced by prominent stochastic components, with any observed trajectory being just one possible realization. Anomalous deviations from a typical maturation path are not merely changes from the observed one, as multiple healthy trajectories might be feasible, and a precise characterization of all such typical trajectories is not known a priori. At the same time, we know that arbitrary perturbations to an observed profile might not be feasible, as evidenced by the challenges of making desired changes to a microbiome profile via various microbial manipulations (*46*). Qualitatively, if two trajectories lead to healthy outcomes, i.e., are exchangeable, then we wish to identify them to be similar, whereas if one ultimately leads to poor outcomes, then our aim here is to be able to recognize the increased clinically meaningful dissimilarity.

With the ability to learn digital twins separately for sub-cohorts corresponding to typical and suboptimal development respectively, we quantify clinically meaningful similarity between two stable microbiome trajectories x, y as the odds of an observed trajectory x being spontaneously replaced by another y by random chance (which is plausible given that observed trajectories are sample paths from underlying stochastic processes). Thus, in our approach, the higher the probability Pr(x → y), the more similar they are. We estimate Pr(x → y) by crafting an intrinsic distance between microbiome trajectories. The *q* distance θ(x, y) between two microbiome trajectories is defined (Definition 3 in Materials and Methods) as the square root of the Jensen-Shannon divergence (*47*) of the conditional relative abundance distributions induced by the inferred Q-net for a specific class at a specific time, averaged over all class–time stamp pairs. We show from theoretical considerations (Theorem 1 in Materials and Methods) that the *q* distance approximates the log-likelihood of spontaneous change in the microbiome trajectories, i.e., θ(x, y) ≈ constant × logPr(x → y).

With this notion of the *q* distance, we can quantify the risk *M*_δ_ of future anomalous deviation for individual patients to address the following question: Given an observed current trajectory of a specific patient (typically with few initial observations), estimate their risk of developing with a cognitive deficit as opposed to proceeding with typical development. We estimate *M*_δ_ risk as the ratio of the log-likelihood of an observed trajectory x (for a given patient) being realized in the suboptimally developing cohort to the log-likelihood of the same trajectory being realized in a typically developing cohort. Thus, our risk estimate quantitatively assesses which of the two models explain the data observed so far for individual patients. To demonstrate the effectiveness of our risk measure, we validate its ability to predict the HCG phenotype in out-of-sample data (the Boston cohort), where the two digital twins corresponding to the typical and suboptimal development, i.e., the AHCG and the SHCG cohorts, are constructed using the UChicago samples.

### Validation of *M*_δ_ risk as a measure of future suboptimal HCG

**Fig. 3.**
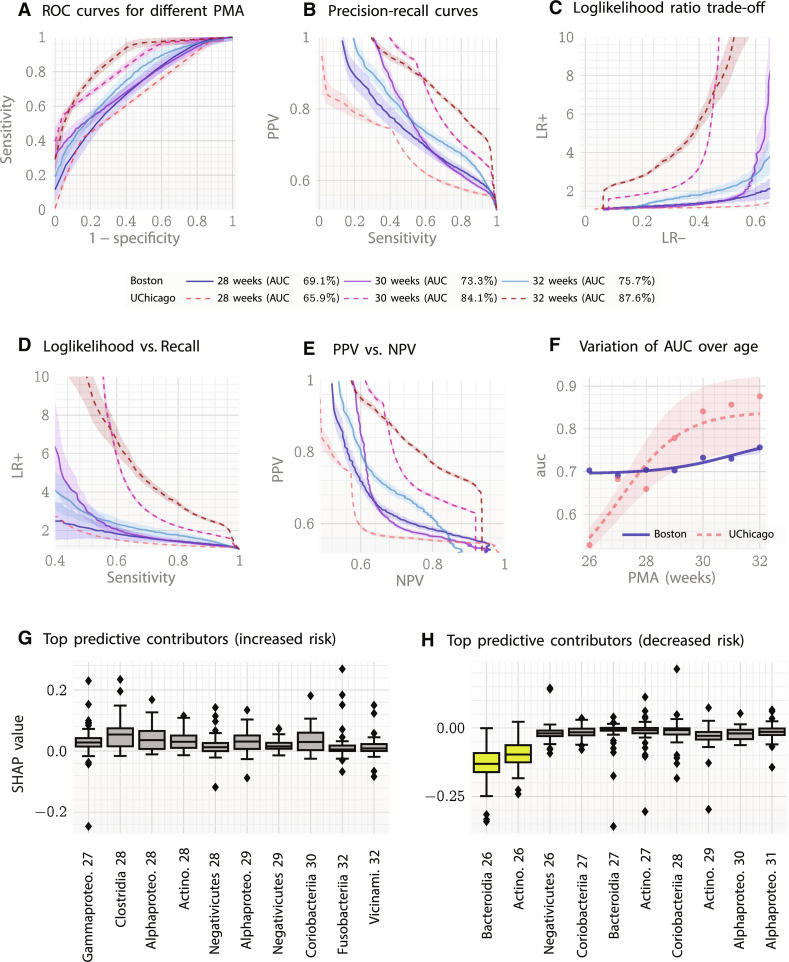
Classification performance out-of-sample. (**A**) Classification performance to recognize infants with eventually suboptimal HCG. The AUC is maximum at 32 weeks PMA reaching 87.6% for the UChicago cohort (in-sample data), and 75.9% for the Boston cohort. (**B**) Precision-recall curves. (**C**) Trade-off between positive (LR+) and negative (LR−) likelihood ratios. (**D**) Change in LR+ with sensitivity or recall. (**E**) Comparison of PPV versus NPV at different PMA weeks. (**F**) Fitting the computed AUCs over time, we note that the AUC > 80% stabilizes approximately over 30 weeks PMA for the UChicago cohort. (**G**) Top positive SHAP values for the *M*_δ_ risk driving HCG classification at 36 weeks PMA, and (**H**) top negative SHAP values for risk. Positive (negative) SHAP values indicate if an individual’s abundance of a specific entity increases (decreases) the risk (compared to baseline samples) of a positive diagnosis of the target disease; thus, the observed levels of Gammaproteobacteria and Clostridia (among others) are often associated with increased individual risk while Bacteriodia and Actinobacteria (among others) are similarly implicated with decreased risk. Note, however, that several taxa appear on both lists, suggesting complex dependencies of risk on entity abundances that vary over time.

To validate our risk measure predicting future anomalous deviations, we set up a classification problem to predict the eventual HCG phenotype of individual patients. We found that classification based on the *M*_δ_ risk yields significantly and substantially improved performance over naive baselines based on microbial relative abundances and clinical factors (baseline model: random forest), as described next. On the UChicago (training) cohort (58 patients, 158 fecal samples, and 18,564 abundance measurements), we achieve peak area under the receiver operator characteristic curve (AUC) at 32 weeks of 87.6% (measured by the out-of-bag score, a proxy for out-of-sample performance; performance achieved using the only *M*_δ_ risk; compared to median AUC 39.3% for a baseline classifier trained using only delivery mode without the risk score; results at different weeks of prediction with additional clinical variables are shown in table S3). At 80% specificity, we achieve a sensitivity of 75.9%, a positive likelihood ratio of 3.79, and a negative likelihood ratio of 0.3, while at 90% specificity, we achieve a sensitivity of 61.7%, a positive likelihood ratio of 6.17, and a negative likelihood ratio of 0.43. On the Boston (validation) cohort (30 patients, 248 fecal samples, and 14,378 abundance measurements), we achieve a peak AUC of 75.7% (performance achieved using the *M*_δ_ risk and the clinical variables delivery mode, birth weight, birth head circumference, and estimated gestational age; compared to a median AUC of 62.4% for a baseline classifier using the same clinical variables but without the risk score) with a sensitivity of 53.3% at 80% specificity, a positive likelihood ratio of 2.67, and a negative likelihood ratio of 0.58 (Fig. 3, A to F). We note that our inferred classifier may be operated with different choice of specificity versus sensitivity trade-offs. For example, we might choose to operate the model at the maximum achievable PPV at a set value of the false-positive rate. The variation of these performance measures at various possible operating points at 30 weeks PMA is enumerated in Table 2, along with 95% confidence bounds. We note that at 2% false-positive rate (98% specificity), we achieve 95 to 96% PPV, and a positive likelihood ratio 17.2 ± 9, which implies that for a patient flagged to be at high risk for developmental deficit, their risk is greater than 9 to 26 times that of the general population. Detailed performance at 32 weeks PMA is shown in table S13. The trade-offs between specificity and sensitivity obtained at different time points measured in weeks PMA is shown in Fig. 3A. Figure 3B shows the trade-offs between positive predictive values (PPVs) and sensitivity, and Fig. 3C shows the trade-offs between the positive and negative log-likelihood ratios. Figure 3F shows the improvement of AUC with patient age, which is expected as more information is collected over time.

**Table 2.** Performance measures for classification at 30 weeks PMA.

UChicago						
fpr	tpr	ppv	acc	npv	LR+	LR−
0.02	0.466 ± 0.015	0.961 ± 0.009	0.714 ± 0.008	0.633 ± 0.004	23.288 ± 0.767	0.545 ± 0.016
0.04	0.541 ± 0.031	0.935 ± 0.009	0.743 ± 0.016	0.661 ± 0.01	13.516 ± 0.773	0.479 ± 0.032
0.06	0.56 ± 0.033	0.909 ± 0.01	0.743 ± 0.017	0.666 ± 0.013	9.328 ± 0.55	0.468 ± 0.035
0.08	0.582 ± 0.029	0.886 ± 0.009	0.745 ± 0.015	0.672 ± 0.011	7.27 ± 0.357	0.455 ± 0.031
0.1	0.598 ± 0.021	0.865 ± 0.007	0.744 ± 0.011	0.676 ± 0.008	5.981 ± 0.206	0.447 ± 0.023
0.12	0.614 ± 0.021	0.846 ± 0.007	0.742 ± 0.011	0.68 ± 0.009	5.116 ± 0.173	0.439 ± 0.024
0.14	0.63 ± 0.02	0.828 ± 0.007	0.741 ± 0.011	0.684 ± 0.009	4.498 ± 0.146	0.431 ± 0.024
0.16	0.645 ± 0.021	0.812 ± 0.007	0.739 ± 0.011	0.688 ± 0.009	4.032 ± 0.134	0.422 ± 0.026
0.18	0.66 ± 0.021	0.797 ± 0.007	0.737 ± 0.011	0.692 ± 0.01	3.665 ± 0.117	0.415 ± 0.026
0.2	0.675 ± 0.023	0.784 ± 0.007	0.735 ± 0.012	0.697 ± 0.011	3.376 ± 0.114	0.406 ± 0.028
**Boston**						
**fpr**	**tpr**	**ppv**	**acc**	**npv**	**LR+**	**LR−**
0.02	0.345 ± 0.181	0.95 ± 0.018	0.651 ± 0.094	0.586 ± 0.069	17.231 ± 9.072	0.669 ± 0.185
0.04	0.367 ± 0.166	0.916 ± 0.02	0.653 ± 0.086	0.589 ± 0.065	9.163 ± 4.156	0.66 ± 0.173
0.06	0.395 ± 0.145	0.887 ± 0.02	0.658 ± 0.075	0.595 ± 0.059	6.588 ± 2.409	0.643 ± 0.154
0.08	0.418 ± 0.124	0.862 ± 0.02	0.66 ± 0.064	0.598 ± 0.052	5.221 ± 1.55	0.633 ± 0.135
0.1	0.441 ± 0.116	0.841 ± 0.019	0.662 ± 0.06	0.602 ± 0.05	4.406 ± 1.165	0.622 ± 0.129
0.12	0.47 ± 0.1	0.821 ± 0.019	0.668 ± 0.052	0.609 ± 0.046	3.917 ± 0.837	0.602 ± 0.114
0.14	0.483 ± 0.094	0.802 ± 0.018	0.665 ± 0.049	0.61 ± 0.044	3.448 ± 0.671	0.601 ± 0.109
0.16	0.5 ± 0.089	0.785 ± 0.016	0.664 ± 0.046	0.612 ± 0.042	3.123 ± 0.555	0.596 ± 0.106
0.18	0.516 ± 0.079	0.77 ± 0.015	0.663 ± 0.041	0.614 ± 0.039	2.868 ± 0.438	0.59 ± 0.096
0.2	0.532 ± 0.078	0.756 ± 0.016	0.661 ± 0.04	0.616 ± 0.04	2.661 ± 0.391	0.585 ± 0.098

### Importance of different taxa in determination of HCG phenotype

To investigate how specific bacterial classes modulate HCG, we perform a Shapley Additive Explanation (SHAP) analysis (*48*, *49*), a model-agnostic method of computing feature importances where the impact of each feature on the model is uncovered using the game-theoretic Shapley values (*50*, *51*). The SHAP analysis (Fig. 3, G and H) found that the top influencers (exerting both positive and negative influences) are most impactful before 30 weeks PMA as evidenced by the time stamps of the top 10 risk increasers (Fig. 3G) and top 10 risk decreasers (Fig. 3H), which are between 28 and 31 weeks PMA. Here, by the standard interpretation of SHAP values, increasing the relative abundance of the microbial classes in Fig. 3G (with positive SHAP values) would increase the risk of a cognitive deficit (and thus these microbes are over-abundant at their corresponding observation times), while those in Fig. 3H (with negative SHAP values) would decrease risk if made more abundant, and are thus under-abundant at their corresponding time stamps. In particular, increasing the 26 weeks PMA levels of Bacteroidia and the 26 weeks PMA levels of Actinobacteria will decrease risk on average, while increasing the 27 weeks PMA levels of Gammaproteobacteria and the 28 weeks PMA levels of Clostridia will increase risk on average. It is notable that the SHAP analysis finds that some of the microbial taxa most contributive to risk, e.g., Negativicutes and Coriobacteriia, exhibit an increasing effect on risk at one time point and a decreasing effect on risk at a different time point, suggesting that Q-nets are capable of capturing subtle variations in the key drivers of risk over time. It is important to note that these average impacts to perturbations are not recommendations that may be used for supplanting the microbiome, as we demonstrate later that such actions necessarily need to be personalized, and what is beneficial for one infant might be detrimental for another.

### Network analysis to uncover across-taxa directional influence on abundance fluctuations

To better interpret the inherently asymmetric directional influences between taxa in the ecosystem, we compute what we refer to as the Local Marginal Regulation (LOMAR) coefficient(s) for each pair of observed interacting bacterial classes. The LOMAR coefficients represent the up-regulatory/down-regulatory influence of a source taxonomic class on a target class, where regulation effects are causally localized in time (future cannot affect the past), and where potential confounding effects from other taxa are averaged out. Using this notion, we can inspect the “influence network” recovered by the Q-net, allowing us to visualize the major differences between the digital twins inferred for the optimal and suboptimal cohorts.

**Fig. 4.**
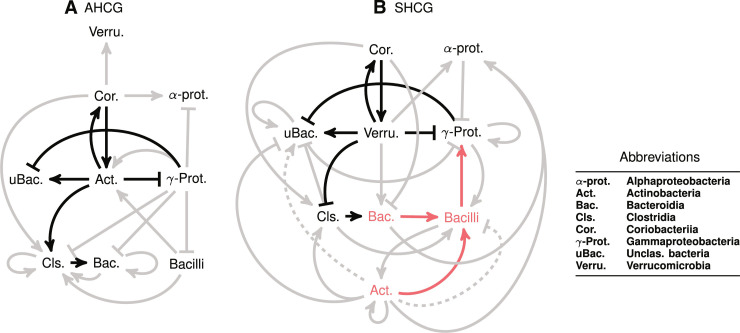
Network structure comparison between typical and suboptimal cohorts. Change in inferred directional dependencies between prominent taxa in early development (≦31 weeks) between optimal and suboptimal HCG, visualized via computing LOMAR coefficients. (**A**) AHCG to (**B**) SHCG. The bold edges highlight some key structural changes in Actinobacteria interactions. The edges in red show that the key changes in Actinobacteria or Bacteroidia are supplanted as potential interventions in suboptimal HCG. Dashed edges for Actinobacteria are interactions that emerge later than their corresponding nondashed ones (tables S5 to S8). Notably, SHCG interactions are both more complex and strongly connected.

After computing the set of LOMAR values for each model (see the “LOMAR of microbial relative abundance” section for details and tables S5 to S8 for the computed LOMAR values), we plot the asymmetric relations directed graphs in Fig. 4. Comparing these networks in early stages of development (27 to 30 weeks PMA, Fig. 4, A and B), we see that the optimal and suboptimal models are quite distinct, with different roles being played by Actinobacteria and Verrucomicrobia as hub nodes, and in general, the SHCG model has more interactions.

### Role of clinical factors and diet in modulating HCG phenotypic outcome

**Fig. 5.**
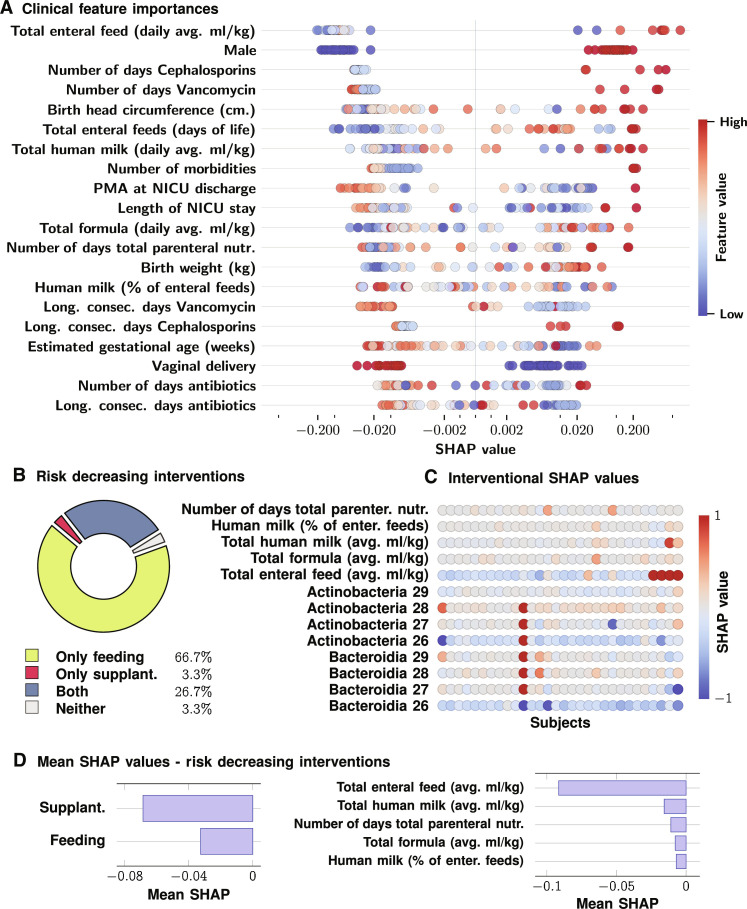
Impact of clinical variables and diet. (**A**) Impact on *M*_δ_ risk of suboptimal HCG. We find that, on average, being male, use of antibiotics, and enteral feed in amount and number of days maximally increase such risk. (**B**) shows the distribution of SHCG patients by the types of intervention found to decrease their risk (microbiome-based supplantation intervention and/or feeding-based intervention). (**C**) depicts SHAP values for variables defining feeding and supplantation interventional categories in the SHCG cohort. (**D**) shows that supplantation features are associated with greater decreases in risk than feeding, but that among feeding interventions, total enteral feed is associated with the maximum decrease in risk.

To assess the importance of clinical factors in modulating microbiome maturation trajectories, we carried out a SHAP analysis of a random forest regression model, with the *M*_δ_ risk as the dependent variable, and all available clinical factors as explanatory variables or features (Fig. 5). We observe that factors such as longer time until total enteral feeding is achieved, sex being male, the total number of morbidities, number of days on Cephalosporins, number of days on Vancomycin, lower gestational age at birth, and total formula in diet substantially raise risk on average. We found vaginal delivery (VD) as the only substantially risk-reducing factor, with a mild benefit from increasing the proportion of human milk in diet. We note that some factors that reduce risk on average increased risk for some participants and vice versa, suggesting the complex contextual impact of these environmental variables. One such example is the total amount of enteral feeds, which highly increased risk of SHCG in a small subset of individuals. Nevertheless, male sex emerges as a key factor driving HCG outcomes.

### Designing personalized interventions to reduce risk of poor HCG outcome

The SHAP analysis of the digital twins inferred for the optimal and suboptimal cohorts lays the foundation for designing early personalized interventions based on supplanting specific microbial taxa in infant diet. Note that a positive SHAP value of a variable (in this case, the relative abundance of a specific taxa at a specific time point) implies that increasing the value of the variable (i.e., the relative abundance of the taxa in question at the particular time point associated with that variable) will increase risk on average, and vice versa. Also note that many of the bacterial taxa appear in Fig. 3 (G and H), i.e., based on the time point, they can be either risk increasing or risk decreasing if their relative abundances are increased. However, not all bacterial taxa have this time-dependent impact, and here we focus on microbial classes that have a consistent average negative SHAP value (i.e., risk-decreasing impact) over time to define effective interventions. Inspecting Fig. 3H, we conclude that Bacteroidia and Actinobacteria are the only two taxa that are substantially and consistently risk decreasing on average, and early supplantation of these taxa is expected to reduce risk of suboptimal outcomes in HCG.

**Fig. 6.**
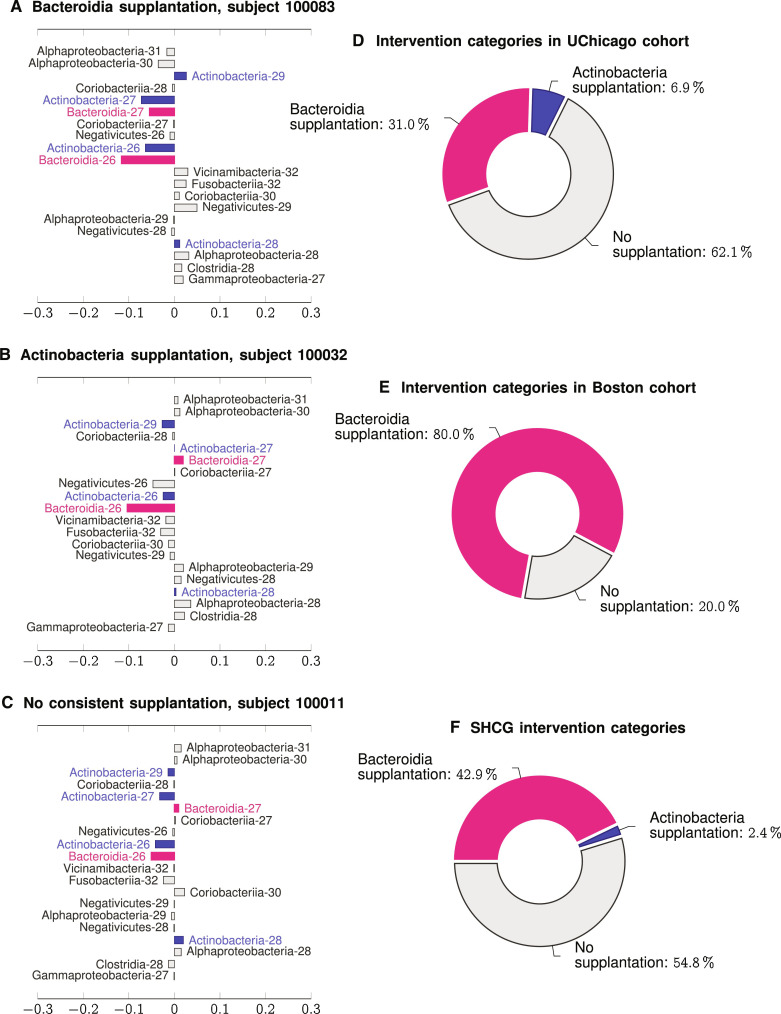
Design of personalized early interventions to reduce risk of suboptimal HCG. (**A** to **C**) SHAP profiles of three patients who have suboptimal HCG, but have three distinct intervention phenotypes, namely, where supplanting Bacteroidia reduces risk (A), supplanting Actinobacteria reduces risk (B), and where no time-independent consistent supplantation can be obtained from our SHAP analysis (C). Both Actinobacteria and Bacteroidia have opposing effects on risk at different time points. (**D** and **E**) The breakdown of these three intervention phenotypes in UChicago and Boston cohorts. (**F**) Breakdown of intervention phenotypes among all patients with suboptimal HCG, showing that 45.2% of patients have discernible interventions.

As before, the average trend is not representative for every participant. Because the SHAP values are patient-specific (Fig. 3, G and H showing averaged values over the UChicago cohort), we can use the notion of consistent impacts as defined above to deduce patient-specific interventions. Using this notion, we end up identifying three intervention phenotypes: (i) Early Bacteroidia supplantation, (ii) Early Actinobacteria Supplantation, and (iii) Null, or patients for whom no such time-independent interventions can be found that would tend to reduce risk. Figure 6 (A to C) illustrates specific patients in these three categories. We found that 31.04% of the UChicago cohort fell into the Bacteroidia supplantation category, with 6.9% in the Actinobacteria category and 62.07% in the Null category (Fig. 6D). The corresponding numbers in the Boston category were 80% amenable to Bacteroidia supplantation, and the rest in the Null category (Fig. 6E). Overall, we found that considering all patients in the suboptimally developing cohorts, 42.9% would have had a risk reduction from early Bacteroidia supplantation, and 2.4% from early Actinobacteria supplantation, while the remaining 54.8% of the patients did not have any time-independent risk-reducing interventions (Fig. 6F). In addition, we found that categories are mutually exclusive, implying that choosing the incorrect intervention could be counterproductive, increasing the risk of suboptimal HCG.

We also investigated such potential intervention designs using the modifiable factors that relate to diet. In particular, we consider the impact of the amount of total enteral feed, total formula fed, total human milk fed, human milk fed as a percentage of total feed, and the number of days the subject received total parenteral nutrition. Examining the SHAP values for these factors (Fig. 5, B and C), we find that the majority of patients (94.4%) with suboptimal HCG have at least one negative SHAP value. Of these, 66.7% do not have a supplantation intervention, while 26.7% may respond to both feeding and supplantation interventions. Thus, our results suggest that most patients with suboptimal HCG may experience possible reduction in risk due to a subset of these specific feeding interventions. While these dietary factors do not appear to have as strong of an effect as supplantation (mean SHAP value of −0.06 versus −0.03 for risk decreasing supplantation samples compared to risk decreasing feeding samples), the feeding interventions are perhaps more broadly applicable. Among the feeding interventions, we find that the greatest risk-decreasing effect from such interventions (on average) appears due to the amount of total enteral feed, which has a mean SHAP of −0.09 compared to ≤ − 0.01 for each of the other variables (Fig. 5D). Note, however, that there is individual variation present (Fig. 5C) with some individuals appearing to have increased risk from this factor, stressing the importance of a personalized, targeted approach to any interventional strategy.

## DISCUSSION

Despite increasing evidence of the critical role of the gut microbiome in pediatric health, the remaining challenges in inferring interactions across diverse microbial taxa obfuscate identification of actionable colonization patterns that might foreshadow poor clinical outcomes. These limitations hinder the design of clinical interventions in vulnerable populations, such as preterm infants, that might otherwise prove effective in ameliorating developmental deficits. Recent work demonstrating the connections between relative abundance of key microbiome taxa and cognitive development in preterm infants (*11*) suggests that subtle patterns in relative abundance data can provide actionable clinical insights. Here, we work toward uncovering these connections via a novel machine learning approach to infer a generative detailed model of the developing ecosystem. Our model is structurally rich, allowing complex time-dependent patterns to emerge, yet sufficiently interpretable to be clinically meaningful.

Considering the problem of uncovering the role of gut microbiota of preterm infants in cognitive development, our key findings are as follows: (i) reliably forecast the coupled development of microbial communities for weeks from very limited initial observations, (ii) obtain clinically useful predictive performance (demonstrated through out-of-sample validation on an external cohort) for prediction of suboptimal phenotypes of cognitive development from microbiome relative abundance profiles, (iii) identify statistically significant direction-specific relationships between entities within the maturing ecosystem, (iv) investigate the relative importance of prominent clinical factors and diet in modulating maturation trajectories, and (v) suggest a principled approach to personalized early interventions that can reduce the risk of suboptimal HCG.

These capabilities are enabled via inferring complex temporal relationships among microbial classes without an a priori fixed parametric structure. Instead of modeling the observed relative abundances to answer specific questions, we learn an approximate yet sufficiently detailed model of multi-way interactions in the ecosystem, yielding a digital twin that enables imputation of missing data, trajectory forecasts, and estimates of future risk of poor developmental outcomes. In addition, our explicit representation of temporal dependencies enables us to identify variations in predictive performance over time, which provide insights into the timing of fundamental shifts in ecosystem development. The ability to extract such digital twins is a substantial improvement over techniques used in the literature, as clearly demonstrated by contrasting our significant outperformance against baseline models such as DBNs. In particular, our ability to replicate high predictive performance in an out-of-sample cohort, comprising patients recruited in a different site, in a different geographical region with very different demographic makeup, provides strong confidence in the robustness of our models and predictions.

It is theoretically possible to use a DBN instead of a Q-net to analyze our data (which we have demonstrated in fig. S2), but it would be inefficient, with poor performance (as we found), for the reason that the DBN would be substantially more complex in structure, there being no reasonable approach to remove edges a priori. Thus, the complexity of inference scales poorly, with the number of edges being quadratic in the number of vertices. The increased number of parameters limits the ability to infer an accurate model from a dataset of fixed size. A standard simplification used by DBN methods (*26*) is to limit the time lag of the model (essentially the temporal memory) to two or three steps. The Q-net scales substantially better, because it requires no such assumptions, and we can easily infer dependencies that may be present across arbitrary time scales, but is substantially less complex, with edges being added more judiciously, leading to better inference of a smaller number of parameters, and in effect, better predictive performance.

Our results and insights here for predicting preterm infant HCG are in many cases extensions of known or suspected results, which provides further corroboration of our findings. For example, we achieve high performance for predicting future HCG phenotype from around 30 weeks PMA, which agrees with earlier work identifying this time point at which developmental trajectories diverge (*11*). However, going beyond known results, we are able to flag individual patients early and reliably for the risk of developmental deficits. While previous work demonstrated a strong correlation of microbial relative abundances with preterm infant HCG after developmental trajectory divergence (*11*), and indeed our approach mirrors these results with substantial differences between microbiome architecture for infants with AHCG versus SHCG, our model extends beyond these findings through leveraging these differences for early predictive diagnoses.

Going beyond patient-specific early predictions of future HCG phenotype, our analysis begins to unravel the complex dynamical processes that drive these outcomes. This is a hard problem, because relative abundance differences across AHCG and SHCG cohorts may not sufficiently explain the differential outcomes. It is well recognized that universal benchmarks for microbial relative abundance ranges are challenging to obtain due to the diversity of microbial communities, which vary significantly between and within individuals over time (*52*), making it difficult to define a universally “healthy” range (*53*) for any taxa. The balance and interaction among different microbial species often play a far more crucial role in determining the health of the ecosystem than their individual relative abundance levels, and factors such as age, diet, environment, and genetics can influence what constitutes a “healthy” microbial community for a particular individual (*54*). We offer a solution to this problem via shifting our focus to how patient-specific perturbations of the relative abundances of specific microbial classes modulate the estimated risk of suboptimal developmental outcomes. Using a standard SHAP analysis of our model, we show that increasing Bacteroidia and Actinobacteria relative abundances in early life is expected to reduce future risk on average, while Clostridia and Gammaproteobacteria increase typically increases risk. Thus, we can conclude that Bacteroidia and Actinobacteria are under-abundant, while Clostridia and Gammaproteobacteria are over-abundant in early life of preterm infants who experience suboptimal HCG. Other microbial classes such as Negativicutes and Coriobacteriia also modulate risk, but do so in a more complex time-dependent and patient-specific manner.

These conclusions are in line with the observations that Bacteroidia was depleted in preterm infants with SHCG and Actinobacteria was further diminished in preterm infants with moderately severe SHCG, as reported on earlier analyses conducted on the UChicago dataset (*11*). The importance of these taxa to infant development has been substantiated through multiple observational and in vivo studies. For example, the relative under-abundance of Bacteroidia has been associated with autism spectrum disorder (*9*) and attention deficit/hyperactivity disorder (*55*). Mechanistically, Bacteroidia may affect neurodevelopment by strengthening intestinal barrier integrity and changing the systemic metabolite profile, as treatment of the maternal immune activation mouse model of autism spectrum disorder with *Bacteroides fragilis* has been directly shown to ameliorate behavioral defects, increase intestinal tight junction protein expression, and alter metabolite levels in the sera (*10*). In addition, the principal genus of Actinobacteria in the infant gut is *Bifidobacterium*, whose relative abundance has been implicated or associated with improved temperamental traits [extraversion (*56*) and soothability (*57*)] and fine motor skills (*58*). In terms of mechanism, treatment of the propionic acid rat model of autism spectrum disorder with *Bifidobacterium longum* has been shown to rescue social deficits and normalize brain-derived neutrophic factor levels in the hippocampal region of the brain (*59*). Our approach, and conclusions, adds to these results by revealing the interactivity of these key taxa within normal and dysbiotic infant gut microbial ecosystems, thus providing the tools for operationalizing these findings via a personalized approach.

While the intricacies of our inferred digital twins are difficult to interpret directly (often an unavoidable artifact of large-scale modeling using predictive analytics), we are able to distill directional dependencies emerging in early life into more simplified networks of key interactions, for AHCG and SHCG cohorts separately, via our LOMAR analysis, which obtain directional up-regulatory and down-regulatory influences between taxa pairs, marginalizing out the impact of other taxa (see Fig. 4). These networks offer an explanation as to why a personalized approach for microbiome interventions is warranted. For example, infants with eventual SHCG that have Coriobacteriia relatively over-abundant in their early microbiomes may not benefit from an intervention with Bacteroidia as the Coriobacteriia can inhibit Bacteroidia proliferation and potentially its engraftment into the ecosystem. Likewise, an Actinobacteria intervention for infants with eventual SHCG that have Clostridia relatively over-abundant in their early microbiomes might be detrimental as the Actinobacteria would promote the high relative abundance of Clostridia. Such network inferences are useful for hypothesis generation of which important infant gut microbial ecosystem interactions should be the subject of future experimental validation.

Finally, we explored the possibility of designing early patient-specific interventions, comprising microbe supplantation. Our strategy in this direction leveraged our patient-specific analysis that reveals how perturbation of specific microbial classes modulates the *M*_δ_ risk. We considered only interventions that are not time dependent, which could be specified simply, e.g., early supplantation of a specific bacterial class, and we ended up with three intervention phenotypes. The first two suggest early supplantation of Bacteroidia and Actinobacteria, the third represent patients for whom no such time-independent risk-decreasing perturbation could be found. The supplant-Bacteroidia phenotype appeared as much more common compared to the supplant-Actinobacteria phenotype, and the *M*_δ_ risk for more than 40% of SHCG patients could actually be reduced in this manner. We found that these phenotypes are mutually exclusive, and the wrong intervention can increase the risk of anomalous maturation of the gut microbiota.

The need for a personalized approach to microbiome intervention in preterm infants is supported by recent randomized controlled trials of probiotic supplementation with *Bifidobacterium* spp. (an Actinobacteria) in neonatal intensive care units (NICUs), which, while demonstrating some benefit for preterm infants in improving intestinal barrier function (*60*) and reducing inflammatory markers in the intestinal milieu (*61*), ultimately reducing the incidence of necrotizing enterocolitis and the length of hospital stay (*62*), have also documented rare adverse events related to probiotic bacteremia (*63*). Furthermore, the efficacy of *Bifidobacterium* spp. probiotic supplementation in the NICU in improving the risk of neurodevelopmental impairment is currently unclear (*64*). Together, these data suggest that although *Bifidobacterium* spp. probiotics will be beneficial for some preterm infants, applying them universally as a prophylactic may not improve neurodevelopmental outcomes overall and can even lead to adverse consequences in certain preterm infants. Our work lends support to these findings, and lays the path to designing additional interventional strategies of Bacteroidia supplantation and enteral feeding that are likely to be more broadly applicable.

These findings underscore the need for high-resolution bacterial profiling in neonatal care. While our results point toward the effectiveness of Actinobacteria or Bacteroidia supplantation in specific patients, the complexity of the neonatal gut microbiome necessitates a more granular bacterial profile that vigilantly exclude potentially pathogenic organisms (*65*, *66*). Despite the limited resolution of the recommendations demonstrated here, our methodology highlights the importance of targeted microbiome interventions and shows that supplantations beneficial for one may be detrimental for another. These results align with the decreasing popularity of the classical one-size-fits-all approach that has shown limitations, as evidenced by mixed outcomes in the universal application of *Bifidobacterium* probiotics in neonates (*67*, *68*). Thus, while we outline a framework for designing safe personalized interventions, further research, particularly in understanding the long-term impacts of these microbial supplantations on neurodevelopmental outcomes in preterm infants, and replicating the results at higher taxonomic resolution, is needed in the future.

Finally, investigating the relative importance of clinical and diet variables, we found that a longer time to achieve total enteral nutrition, the use of antibiotics, being male, and not having a VD substantially increased the risk of anomalous deviation in microbiome maturation trajectories (Fig. 5). These findings, as before, are well corroborated in the literature (*69*–*73*). The absolute impact of these variables, with the exception of infant sex, were small, complementing previous work on preterm infants that demonstrated that the dispersal of microbes in the limiting NICU environment supersedes the effects of habitat filtering factors such as diet and antibiotics in shaping microbiomes (*11*). It is also important to note that the *M*_δ_ measure we propose here makes precise prediction based on microbiome profiles, demonstrated to be affected marginally by diet and environmental and clinical variables, underscoring the potential utility of our approach. As an example, we found that most preterm infants would benefit from increased enteral feeding amounts as expected, but the reduction in risk of SHCG was not as impactful as microbiome supplantation, and further, there was a smaller subset of infants for which increasing enteral feeding amounts would greatly increase the risk of SHCG. Feeding intolerance of preterm infants has been linked to dysbiosis of the gut microbiome (*74*, *75*), and certain randomized controlled trials of probiotic administration to preterm infants in the NICU demonstrate decreased incidences of feeding intolerance (*76*–*78*). Therefore, our methodology is additionally able to resolve when non–microbiome-based interventions may be more appropriate, and also when such interventions would likely be inappropriate given the underlying microbiome structure of the infant such as ramping up enteral feeds.

Our study had some limitations. We were unable to ascribe a potential preventative intervention that could substantially reduce risk of SHCG for a portion of preterm infants among both UChicago and Boston cohorts. This finding is not unexpected as neurodevelopment is multifactorial with certain risk drivers likely being independent of the microbiome and thus were not considered in this study. Examples of such drivers include but are not limited to intrauterine growth restriction or maternal health, and respiratory, cardiovascular, or eye diseases (*79*, *80*). Furthermore, while our results were well replicated in two different sites, we did not primarily aim to investigate site-specific effects and the effects of all clinical variables or to compare cohorts. Future work could investigate the layered influence of clinical factors using larger datasets from multiple sites that would contain more variation in clinical variable combinations. Furthermore, it would be of interest to follow microbiome maturation and neurodevelopment of infants after NICU discharge, as the home environment might alter the relative impact of clinical variables on microbiome trajectories compared to the NICU environment. The NICU stay is but one part of the critical time window of preterm infant neurodevelopment, and although HCG in the NICU is strongly correlated with later infant outcomes (*35*), phenomenon such as catch-up growth or later growth failure has been reported after NICU discharge (*32*). The Q-net could be constructed for longer time courses in the future to solve these knowledge gaps for revealing key time points and risks of microbiome trajectory deviation throughout development. The scalability of the algorithm suggests that we can explore the gut microbiota at lower taxonomic levels or use more detailed clinical factors (e.g., donor milk versus mom’s own milk and use of fortifiers and supplements for enteral feeding) to obtain sharper interventional prescriptions. In addition, we note that larger sample sizes, with more longitudinal observations per participant, will also help to further characterize uncertainty and robustness properties of the inferred models.

Using the Q-net framework, we demonstrated clinically interesting performance in reconstructing digital twins of healthy and dysbiotic microbiome maturation trajectories for prediction of infant developmental outcomes, despite the relatively small number of patients and samples. Nonetheless, application of larger datasets in the future will examine microbiomes at finer taxonomic resolutions where there is more variability among subjects. Furthermore, larger-cohort clinical and experimental validation of our modeling framework is warranted to establish robust utility in predictive diagnostics and in designing therapeutic interventions, respectively. Ultimately, this study lays the foundation of a new set of tools to analyze relative abundance data, with the possibility of future predictive screening for serious disease from microbial profiles and designing precise yet effective clinical interventions.

## MATERIALS AND METHODS

### Data sources

To test our approach, we utilize microbiome profiles obtained from cohorts of (i) 58 preterm infants born less than 35 weeks gestational age recruited (*11*) (see Table 1) from University of Chicago’s Comer Children’s Hospital, and (ii) 30 preterm infants born less than 35 weeks gestational age recruited from the Beth Israel Deaconess Medical Center (Boston, MA) as part of the MIND study. Longitudinal fecal samples were sent for genomic DNA extraction and Illumina 16*S* rRNA gene sequencing. (*38*, *39*) Data were processed and merged by the sample inference tool DADA2 (*81*) using QIIME2 version 2019.7 (*82*), then classified to the genus level by the IDTAXA method (*83*) with the R package DECIPHER version 2.14.0 using the Genome Taxonomy Database (*84*) version 89, and additionally into species-like groups by the online NCBI Nucleotide Basic Local Alignment Search Tool (*85*) (BLAST) with an identity threshold of ≥97%. After classification, low-quality samples with <1000 total sequence counts were removed, and then species-like groups that represented <0.1% mean abundance were culled. We utilize the relative abundances of microbes from these samples (taken at the taxonomic level of Class, though we note that our approach can be applied at arbitrary—even mixed—taxonomic levels).

### Definitions and notation

We describe the details of Q-net construction and inference in a general context. The Q-net is a model of the ecosystem structure present in collections of mutually dependent discrete (or discretized) features, such as quantized microbiome relative abundance profiles. The Q-net explicitly estimates individual conditional distributions of each feature (which collectively serve as a model of the full joint distribution of the ecosystem).

**Definition 1** (Q-net). *Let X~P be an n*-*dimensional discrete random vector supported on a finite set* Σ *and following distribution P*, i.e.

$$X=\left(X_{1},\ldots ,X_{n}\right)∼P,\;\text{supp}\left(X\right)=\Sigma =\prod_{i=1}^{n}\;\Sigma_{i}\;\text{with}\;|\Sigma |<\infty$$

For *i* = 1, …, *n*, *let P_i_* ≔ *P*(*X_i_*∣*X_j_* = *x_j_ for j* ≠ *i*) *denote the conditional distribution of X_i_ given the values of the other components of X*. *Finally, for each i* = 1, …, *n*, *let* $\Phi_{i}^{P}$ *denote an estimate of the distribution P_i_*. *Then, the set* $\Phi^{P}≔{\left\{\Phi_{i}^{P}\right\}}_{i=1}^{n}$ *is called a* Quasinet (Q-net) *for the population P*.

When *P* is clear from context, we may omit the superscript and simply write Φ = {Φ*_i_*} to denote the Q-net. The motivation for Definition 1 is that the collection of all estimators Φ = {Φ*_i_*} contained in a Q-net represents the set of all inferred dependencies from the observed ecosystem. While the definition allows for arbitrary method of algorithm to construct the estimators Φ*_i_*, the utility of a Q-net clearly depends primarily on the properties of the Φ*_i_*. Here, we aim to minimize the set of a priori assumptions on the overall model structure to allow the complex dependencies present in *P* to emerge. To that end, throughout this work, all Q-nets are computed using conditional inference trees (*42*) (a variant of classification and regression trees) to compute each Φ*_i_*. In general, each Q-net component Φ*_i_* is computed independently from the other Φ*_j_*, which allows a network structure to form among these estimators. Given a Q-net Φ, it is of interest to determine intrinsically how well Q-net represents the data. Here, we define an explicit model membership test to address this.

**Definition 2** (Membership Probability). *Given a population P inducing the Q-net* Φ*^P^ and a vector* x = (*x*_1_, …, *x_n_*), *the membership probability of* x *in the set of samples modeled well by the Q-net*

$$\omega_{x}^{P}≔\text{Pr}\left(x\in P\right)=\prod_{i=1}^{n}\;\Phi_{i}^{P}\left(X_{i}=x_{i}|X_{j}=x_{j},j\neq i\right)$$ (1)

*which represents the probability that the Q-net generates the sample* x.

We can assess the goodness of fit of an inferred Q-net by testing if the null hypothesis *H*_0_: “samples have a higher probability of being generated by randomly selecting responses, compared to being generated by the inferred Q-net” is rejected.

The Q-net allows us to rigorously compute bounds on the probability of a spontaneous change from one vector to another, induced by chance variations. Not all perturbations in a vector are either likely or biologically meaningful. With an exponentially exploding number of possibilities in which a vector over a large set of items can vary, it is computationally intractable to directly model all possible dynamics; nevertheless, we can constrain the possibilities using the patterns distilled by the Q-net construction. A key piece of this approach is to design an intrinsic distance between vectors, which is reflective of the underlying biological structure of the network.

**Definition 3** (*q* distance). *Let* $\Phi^{P}={\left\{\Phi_{i}^{P}\right\}}_{i=1}^{n}$ *and* $\Phi^{Q}={\left\{\Phi_{i}^{Q}\right\}}_{i=1}^{n}$ *denote Q-nets on the distributions P and Q*, *and suppose* x = (*x*_1_, …, *x_n_*) *and* y = (*y*_1_, …, *y_n_*) *are samples of X*~*P and Y*~*Q_,_ respectively. Then, the q distance* θ_*P*, *Q*_(x, y) *between* x *and* y *is*

$$\theta_{P,Q}\left(x,y\right)≔\frac{1}{n}\sum_{i=1}^{n}\;\left[J^{\frac{1}{2}}\left(\Phi_{i}^{P}\left(X_{i}|X_{j}=x_{j},j\neq i\right)∥\Phi_{i}^{Q}(Y_{i}|Y_{j}=y_{j},j\neq i\right)\right]$$

*where* $J^{\frac{1}{2}}$ *denotes the Jensen-Shannon distance induced by Jensen-Shannon divergence* (*86*).

For brevity, we may write θ*_P_* instead of θ_*P*,*P*_ or simply θ if the distribution(s) are clear from context. Because the Jensen-Shannon distance $J^{\frac{1}{2}}$ is a metric (*87*) on the set of probability distributions, and θ inherits nonnegativity and symmetry, and respects the triangle inequality, it follows that *q* distance is a (pseudo)-metric on Σ. Note that, being a pseudo-metric implies that we may have θ(x, y) = 0 for x ≠ y; i.e., distinct vectors can induce the same distributions over each index, and thus have zero distance. This is in fact desirable, because we do not want our distance to be sensitive to changes that are not biologically relevant. The intuition is that not all variations are equally important or likely. Moreover, we show in Theorem 1 that the log-likelihood of a vector x transitioning to y scales with *a*(x, y), allowing us to directly estimate the probability of spontaneous (or sequential) jumps between relative abundance profiles.

The ability to estimate the probability of spontaneous jump between relative abundance profiles in terms of θ has crucial implications for our study of evolving microbial ecosystems, as it gives us the ability to simulate realistic forecasts of microbial evolution from any given initial profile and generate estimates of the risk of occurrence of a suboptimal clinical phenotype.

**Theorem 1** (Probability Bound). *Given a vector* x *of length n from P that transitions to* y *from Q*, *we have the following bounds at significance level* α

$$\omega_{y}^{Q}e^{\frac{\sqrt{8}N^{2}}{1-\alpha }\theta \left(x,y\right)}≧\text{Pr}\left(x\to y\right)≧\omega_{y}^{Q}e^{-\frac{\sqrt{8}N^{2}}{1-\alpha }\theta \left(x,y\right)}$$ (2)

*where* $\omega_{y}^{Q}$ *is the membership probability of* y *in the population Q (Definition 2), and* θ(x, y) *is the q distance between* x, y *(Definition 3)*.

**Remark 1**. *This bound can be rewritten in terms of the log-likelihood of the spontaneous jump and constants independent of the initial sequence* x *as*

$$|\text{log}\text{Pr}\left(x\to y\right)-C_{0}|≦C_{1}\theta \left(x,y\right)$$ (3)

*where the constants are given by*

C0=log ωyQ (4)

C1=8N21−α (5)

Theorem 1 gives theoretical backing to the claim that samples generated by the Q-net indeed reflect likely perturbation possibilities from the current state. Thus, we can use the Q-net to draw biologically realistic samples that respect the underlying feature dependencies arising from the constraints of the underlying network (that is, the Q-net-inferred conditional distributions can be used to approximately generate samples from the population *P*). This has several implications, such as the ability to easily handle missing/incomplete data.

### Q-net construction from relative abundance profiles

In applying the Q-net construction to microbiome data, we use sample data *D* = {x*_k_*∣*k* = 1, …, *d*} consisting of a set of *d* = 58 patient vectors of the form x*_k_* = (*x*_*k*1_, …, *x_kn_*), where *x_kj_* denotes the relative abundance of the *j*th entity in the *k*th sample. Because the samples are longitudinal measurements of the patients, some of the *x_kj_* represent measurements of the same microbial entity at different time points in the *k*th patient’s observed history. Thus, in this case, Σ represents the quantized relative abundances of all measured microbiome entities across all weeks and each sample x*_k_* consists of all relative abundances of across all weeks for a given individual. Note that we do not impute or fill in missing observations when constructing the Q-net.

Again, while the Q-net allows for essentially arbitrary choice of features, including the ability to specify different taxonomic levels for different entities in its construction, here we fix all entities at the Class level as described before. To deal with the compositional nature of microbial abundance data, we first quantize the relative abundance estimates into a finite number of bins corresponding to quantiles of the range of each entity-specific relative abundance. This step transforms ratios of observations to categorical variables that interpret magnitudes in terms of how they are distributed over the population distribution, and thus are substantially more stable. More precisely, if β*_t_* denotes the set of all observations of a particular microbial taxa at time *t*, then for quantization of β*_t_* into *k* bins, we first fix a bin width of Δ ≔ 1.002 ⋅ range(β*_t_*)/*k*. Letting *m*_∗_ = min (β*_t_*) − 0.001 ⋅ range(β*_t_*), we then quantize observations via the mapping

$$\psi :\beta_{t}\to \left\{A_{1},A_{2},\ldots ,A_{k}\right\}$$

defined by ψ(*b_t_*) = *A_i_* if and only if *b_t_* ∈ (*m*_∗_ + (*i* − 1)Δ, *m*_∗_ + *i*Δ]. For specificity, we often use *k* = 26 throughout our computations (aiming to have a high quantization resolution) and utilize the standard letters of the alphabet A, …, Z as categorical labels in place of *A*_1_, …, *A*_26_. A graphical example of the quantization ranges computed for several key microbial taxa is shown in fig. S3. The model and its subsequent inference tasks operate directly on these quantized (categorical) data; however, for some applications, it is desirable to convert from quantized to numeric relative abundances; for this dequantization, we return the midpoint of the corresponding interval used in the mapping ψ.

### Q-sampling: Efficient high-dimensional sampling of the Q-net

From the Q-net, we have inferred approximations to the full conditional distributions. As the collection of full conditionals can be shown to uniquely determine the full joint distribution [by the Hammersley-Clifford theorem (*88*)], we can then take the Q-net approximations as a model of the joint distribution. Specified in this form, we obtain an efficient method of sampling the (high-dimensional) model.

In particular, while it would be computationally difficult to directly sample a model distribution over hundreds or thousands of variables, we leverage the inferred conditionals in a natural way. In particular, starting from a known sample, we may iteratively update its indices by sampling the corresponding conditional distribution in the Q-net. We then proceed to sample the next index, now using the value generated in the previous step.

This procedure can be used to both generate new, realistic samples reflecting the model dependencies and impute missing values that may be present in the data. To clarify, suppose the *k*th sample is x*_k_* = (*x*_*k*1_, …, *x_kn_*). We may define an indicator of missing values m*_k_* = (*m*_*k*1_, …, *m_kn_*), where *m_kj_* = 1 if *x_kj_* is missing and *m_kj_* = 0 otherwise. The q-sampling routine is then:

1. Choose an index *j* ∈ {1, …, *n*} for which *m_kj_* = 1 and impute feature *x_kj_* by sampling the distribution Φ*_k_*(*X_k_*∣*X_i_* = *x_i_*, *k* ≠ *i*).

2. Go back to step 1, until no unobserved entity remains.

This kind of sampling algorithm is not entirely unprecedented. Schematically, this procedure is similar to the well-known Gibbs sampling routine (*43*, *89*), which also uses iterative samples from full conditional distributions to generate samples from the joint distribution asymptotically. However, unlike Gibbs sampling, q-sampling uses fixed approximate conditional distributions inferred by the Q-net and initializes from a known sample, which can allow q-sampling to converge faster (without the burn-in period often observed with arbitrary initialization of Gibbs sampling).

### *Q* distance induced risk stratification

Here, we use the *q* distance to define a risk measure* M*_δ_ to track anomalous deviations from typical microbiome maturation.

**Definition 4**. *Suppose P and Q are distributions with common finite support* Σ *and inducing Q-nets* Φ*^P^ and* Φ*^Q^. Let* x = (*x*_1_, …, *x_n_*) *denote a sample realization of X* = (*X*_1_, …, *X_n_*) *where X is a discrete random variable with* supp(*X*) = Σ*. Let* 0*_P_ denote a vector sampled from P with all features missing; similarly, we denote* 0*_Q_. Then, we define the P-risk of* x *as*

$$A_{\delta }\left(x\right)=\frac{\theta_{P}\left(x,0_{P}\right)}{\theta_{Q}\left(x,0_{Q}\right)}$$

In view of Theorem 1, the *P*-risk is analogous to a likelihood ratio; high values of the *P*-risk indicate that x is more likely to have been generated by *P*. Here, we take *P* to represent the clinical cohort that attains suboptimal HCG and *Q* to represent the group attaining optimal HCG.

### Risk-based prediction of suboptimal cognitive development

To use the risk measure *A*_δ_ for classification of clinically suboptimal phenotype, we first compute the corresponding Q-nets Φ*^P^* and Φ*^Q^* for the suboptimal and appropriate HCG cohorts, respectively. From this, we can compute the *P*-risk of each sample x.

We expect that infants with higher values of *A*_δ_(x) have a higher risk of developing suboptimal HCG. It is in fact possible to generate a classifier that performs well (in-sample) by fixing a decision boundary on the value of *A*_δ_. However, to determine how informative *A*_δ_ is as a part of a possible clinical screening tool, we train a random forest classifier using *A*_δ_ and a limited selection of clinical factors known at the time of birth (for the UChicago cohort, we use only the delivery mode; for the Boston cohort, we also include birth weight, birth head circumference, and gestational age). For these classifiers, we then assess the median AUC as a function of the week of classification, with *A*_δ_ computed using only the values observed before the specified week. Then, to assess the impact of individual bacterial classes on the risk, we perform a standard SHAP analysis, estimating the Shapley values of each *x_i_* using the Kernel SHAP (*48*) method.

### Forecasting coupled microbiome maturation

As our features correspond to relative abundance measurements at a given time point, we can generate forecasts of the variation of relative abundance of a specific class over time. When forecasting ecosystem maturation either on average among a host population or within individual hosts, we must only apply causal rules in computing the predictions (i.e., we must not use features with values in the future). The Q-net framework makes applying this restriction straightforward. We assume a period of initial observation (say 4 to 8 weeks) and fix a time *t* from which to initiate forecasting. Then, in the desired individual or cohort, we mask out all features corresponding to times >*t* (treating the corresponding missing variable indicators *m_kj_* as unity). We then iteratively sample the Q-net to get estimates of relative abundance levels at a particular time, proceeding as follows:

• Choose randomly an index from the indices corresponding to time *t* for which the indicator *m_kj_* = 1; generate a q-sampling from the corresponding distribution at time *t* − 1;

• Go back to step 1 and choose a new index, unless all missing values at time *t* have been imputed

Once all observations at this time step *t* have been obtained as above, we move to the next time step *t* + 1, assuming that all prior observations are now available.

To evaluate the efficacy of this approach to forecasting using Q-nets, we compared results attained to an approach using DBNs. For this experiment, DBNs of model depths 2, 3, 4, 5, and 6 were constructed from the UChicago data. Models were fit using extttR (*90*) version 4.1.1 with packages dbnR (*91*) and bnlearn (*92*). Using the models trained on the UChicago cohort, forecasts were generated from 28 and 31 weeks, respectively, from initial conditions specified by the time lag of the model under both the UChicago (in-sample forecasting) and Boston (out-of-sample forecasting) cohorts. For DBNs of depths 4 to 6, 28-week forecasts are not generated because the time lag prevents proper specification of initial conditions (we only utilize relative abundance measurements occurring from ages ≥25 weeks PMA).

### LOMAR of microbial relative abundance

For any given entity *X_i_*, the Q-net provides a natural estimate of the conditional dependence on other entities via the inferred predictor Φ*_i_*. By examining the specific/localized pairwise interactions implied by these dependencies, we may further use these predictors to generate estimates of the marginal change in relative abundance of *X_i_* (the “target” entity) resulting from changes to the relative abundance of another entity *X_j_* (the “source” entity). As the relative abundances are real-valued and the Q-net’s distributions are discrete (categorical), we must both marginalize out other entities and pass to a continuous distribution to generate these estimates.

Specifically, we first marginalize out all other entities from Φ*_i_* to generate the conditional response as a function of *x_j_* alone, i.e., $\Phi_{i}^{j}\left(X_{i}|X_{j}=x_{j}\right)$ (recall this is a discrete distribution supported on Σ*_i_*). We then de-quantize the support of *X_i_*, generating a discrete distribution supported on [0,1] (relative abundance values).

To identify the marginal contribution of small perturbations of *X_j_* to the relative abundance of *X_i_*, we would like to estimate the derivative of the unknown regression function *f*(*x_j_*) ≔ *E*[*X_i_*∣*X_j_* = *x_j_*] (analogous to fitting a local linear regression model to *f*); however, rather than samples near *x_j_*, we have a (discrete) distribution $\Phi_{i}^{j}$ , which is an approximation of the true continuous distribution. To generate additional samples, we then draw from $N\left(\mu_{i}^{j},\sigma_{i}^{2j}\right)$ , where $\mu_{i}^{j}$ and $\sigma_{i}^{2j}$ denote the sample mean and variance of the discrete distribution.

### Q-net model regeneration

Because our approach to determining the Q-net dependency structure is probabilistic, we can empirically estimate the variability of our approach. In particular, for each result, the analytical pipeline was regenerated in accordance with table S16 to assess consistency/robustness of the stated results.

### Software availability

To facilitate Q-net–based analysis of microbiome data, we developed a Python package, which is installable from PyPi (https://pypi.org/project/qbiome/). The package includes functions for constructing models and performing analysis of longitudinal datasets. We have written example notebooks demonstrating basic utilization of the package—including model construction and quantization of relative abundance data, which are available in the package repository (https://github.com/zeroknowledgediscovery/qbiome/tree/main/examples). These notebooks demonstrate the usage of the package to perform analytical methods utilized above, and are also made available at https://github.com/zeroknowledgediscovery/qbiome/tree/main/examples/publication_examples; in particular, these demonstrate the following:

1. Forecasting of microbiome trajectories (https://github.com/zeroknowledgediscovery/qbiome/tree/main/examples/publication_examples/forecasting_trajectories/generate_forecasts_trajectories.ipynb)

2. Computation of *M*_δ_ (https://github.com/zeroknowledgediscovery/qbiome/tree/main/examples/publication_examples/risk/compute-risk.ipynb)

3. Computation of LOMAR coefficients (https://github.com/zeroknowledgediscovery/qbiome/tree/main/examples/publication_examples/lomar/compute-lomar.ipynb)

4. Computation of SHAP values to identify possible patient-specific microbiome interventions (https://github.com/zeroknowledgediscovery/qbiome/tree/main/examples/publication_examples/shap/compute-shap-profiles-phenotypes.ipynb)

5. Computation of SHAP values to identify possible patient-specific clinical interventions (https://github.com/zeroknowledgediscovery/qbiome/tree/main/examples/publication_examples/clinical_shap/shap-clinical-factors.ipynb)

For these examples, we provide prequantized relative abundance data from the UChicago and Boston cohorts; when applying to new data, users may utilize the provided quantization methods to quantize data for analysis.

### Ethics statement

Data collection and research were reviewed and approved by the University of Chicago Institutional Review Board with IRB number IRB16-1431, covering both sites. Participating subjects were enrolled after receiving written informed consent from the subject’s legal guardian, and the language of the consent form was approved by the IRB of record.

## Supplementary Materials

### This PDF file includes:

Figs. S1 to S3 Tables S1 to S16 References
