# Supplementary material for: A digital twin of the infant microbiome to predict neurodevelopmental deficits
Source: Sci Adv. 2024 Apr 10;10(15):eadj0400. doi: 10.1126/sciadv.adj0400 (PMC11006218; doi:10.1126/sciadv.adj0400)
Supplement: Supplementary file 1 — Figs. S1 to S3 Tables S1 to S16 References [file sciadv.adj0400_sm.pdf]

Science Advances
AAAS

Supplementary Materials for

A digital twin of the infant microbiome to predict neurodevelopmental deficits

Nicholas Sizemore *et al.*

Corresponding author: Ishanu Chattopadhyay, ishanu@uchicago.edu

Sci. Adv. **10**, eadj0400 (2024)
DOI: 10.1126/sciadv.adj0400

This PDF file includes:

Figs. S1 to S3
Tables S1 to S16
References

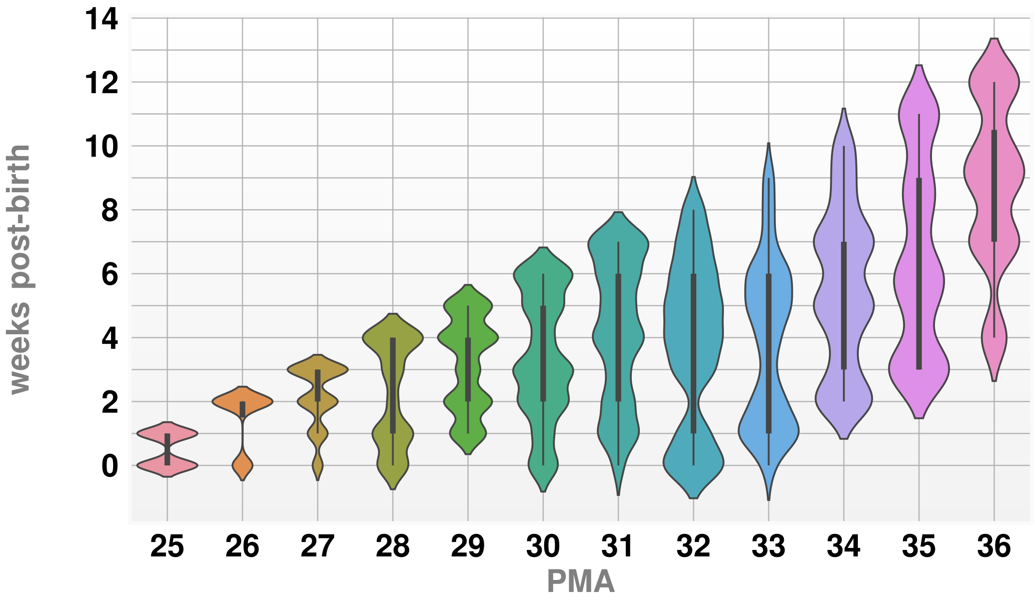

Fig. S1: Distribution of age measured in weeks post-birth for various ages measured in weeks postmenstrual age

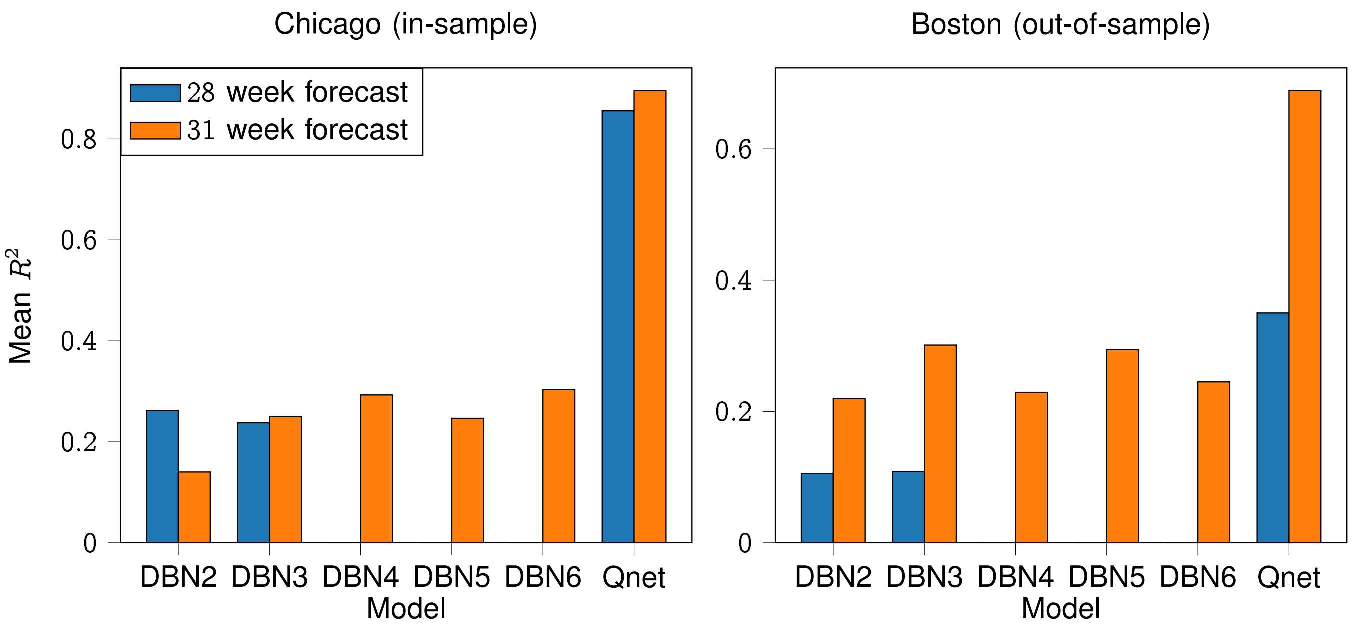

Fig. S2: **Comparison of forecasting performance between Q-nets and Dynamic Bayesian Networks.** Performance is assessed via R^2 between forecasted and actual relative abundances across the key taxa shown in Figure 2 between Dynamic Bayesian Networks of stages 2-6 and the Q-net (for stages $4-6$, 28 week forecasts are unavailable due to insufficient initial conditions). For each cohort, the Q-net achieves a mean R^2 at least twice that of a DBN of any depth for both the 28 and 31 week forecasts.

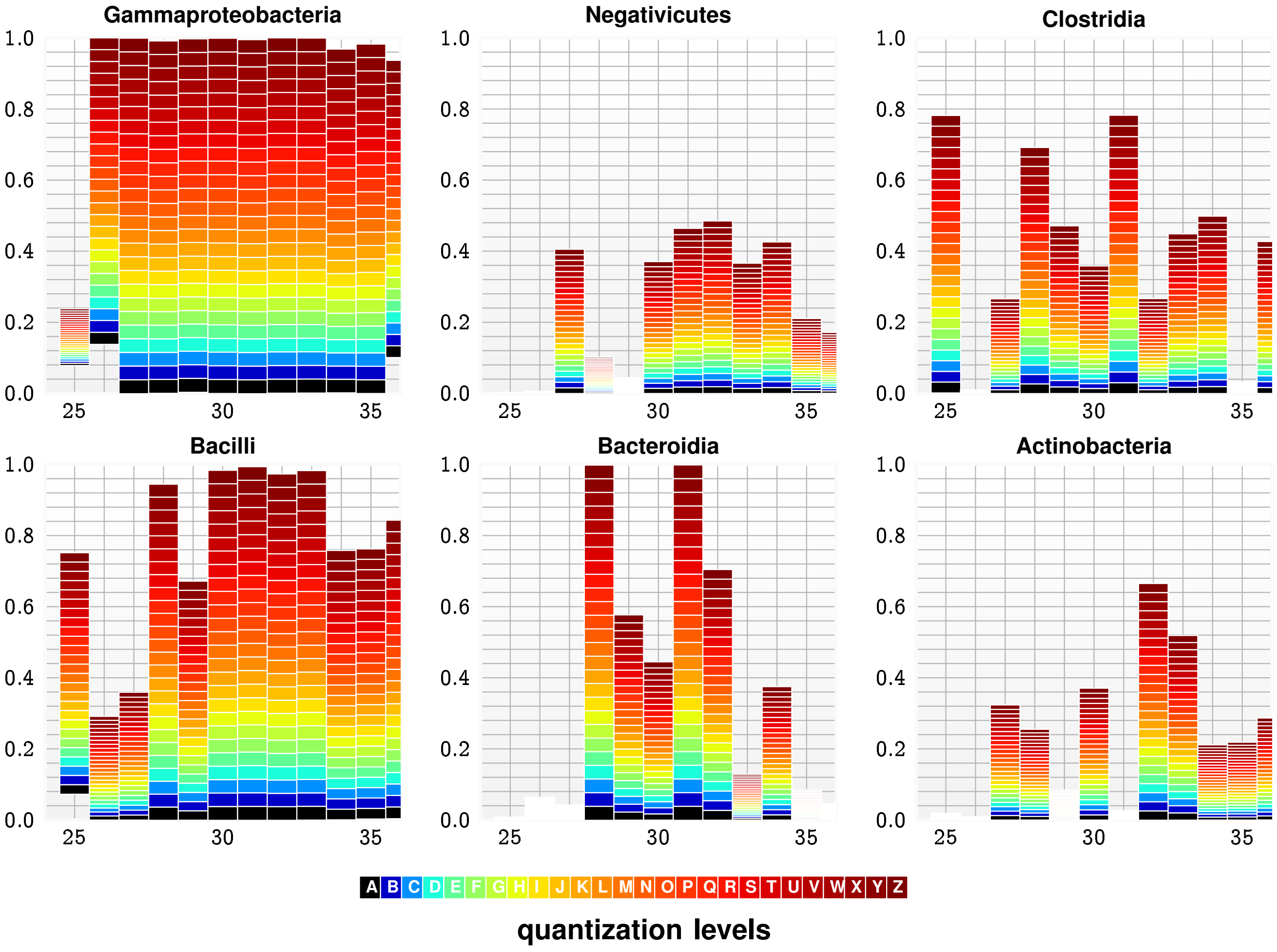

Fig. S3: **Visualization of the quantized ranges of relative microbial abundances.** For each microbial taxa, the quantization mapping (with 26 bins) was applied separately to weekly observations of several key microbial taxa. Note that the ranges for some weeks are too small to visualize clearly.

TABLE S1: Studies relating cognitive, developmental or neuropsychiatric disorders to the gut microbiome

Year	Disorder Studied	Citation	Sample Size	Major Bacterial Taxa	Taxonomic Class*†	Long. analysis
2013	Autism	Kang et al. [9]	20	Prevotella	Bacteroidia†	No
2013	Autism (Mouse Mod.)	Hsiao et al. [10]	N/A	Bacteroides fragilis	Bacteroidia†	No
2016	Alzheimer's Disease	Vogt et al. [93]	25	Firmicutes, Bacteroidetes	Clostridia†, Bacteroidia*	No
2016	Multiple Sclerosis	Jangi et al. [94]	60	Methanobrevibacter, Akkermansia	Euryarchaeota*, Verrucomicrobiae*	No
2017	Major Depressive Disorder	Jiang et al. [95]	76	Lachnospiraceae, Ruminococcaceae	Clostridia†	No
2020	Attention Deficit Hyperactivity Disorder	Prehn-Kristensen et al. [55]	14	Bacteroidaceae, Prevotellaceae	Bacteroidia†	No

* Overabundance † Underabundance

TABLE S2: Comparison of existing models used for forecasting microbial dynamics.

Reference	Model	Comments	Limitations	Training Data	Validation Data	Number of Taxa/Samples	Results
MDSINE [29]	Generalized Lotka-Volterra (ODE)	novel Bayesian methods for parameter estimation	Models only pairwise interactions; linear approximation of gLV dynamics	Actual: 1) n=5 gnotobiotic mice; 2) n=17 germ-free mice	None	1) 13 taxa (Species); 2) 17 strains (Clostridia)	RMSE = 0.56 (measured in CFUs/g stool)
CGBayesNet [25]	Dynamic Bayesian Network	Conditional Gaussian Bayesian model; joint distribution normal mixture	Markov assumption; assume fixed number of ”parents” for each node	Actual: n=58 preterm infants (gut)[23]	None	29 taxa (Class); 922 samples	MAE (actual vs. forecasted, per subject): ≈ 0.04
SgLV-EKF [30]	Generalized Lotka-Volterra with Extended Kalman Filter	directly incorporates filtering of experimental measurements	Assume noise is normally distributed	Actual: n=2 mice (gut)[96]	None	11 taxa: 10 (Genus), 1 (Species)	Qualitative agreement of forecasted abundance levels
Gibson, T. et. al[24]	Generalized Lotka-Volterra; Bayesian nonparametric	stochastic gLV dynamics with uncertainty propagation and clustering of microbes into “interaction modules” to reduce number of inferred interactions	No interactions assumed within modules; models only pairwise interactions and quadratic nonlinearities	Actual: n=5 gnotobiotic mice[29]; Synthetic: n=5 subjects simulated from cluster model	None	Actual: 13 taxa (Species); 130 samples Synthetic: 13 taxa (Species); 55 samples	RMSE (simulated vs. forecasted abundance) = 1.88

Continued on next page

Reference	Model	Comments	Limitations	Training Data	Validation Data	Number of Taxa/Samples	Results
Gao et al. (2018)[31]	Generalized Lotka-Volterra	forward stepwise regression and bagging to fit model parameters	Models only pairwise interactions	Actual: n=1 cheese community [97]	None	9 taxa (Species)	RMSE (actual vs. forecasted relative abundance, mean per taxa): $= 0.054$
Lugo-Martinez et al.[26]	Dynamic Bayesian Network	temporally aligns microbiome profiles before model construction	Markov assumption; assume fixed number of "parents" for each node	Actual: 1) n=58 preterm infants (gut)[23]; 2) n=32 adult women (vaginal)[22]; 3) n=18 pregnant women (oral cavity) [98]	None	1) 29 taxa (Class); 2) 330 taxa (Species); 3) 374 samples	MAE (per taxon, weekly sampling): 1) 0.99; 2) 1.28; 3) 2.18
LUMINATE[27]	Hidden Markov Model	includes explicit model of technical noise (measurement error)	Markov assumption	Synthetic/Actual: 1) simulated/actual samples from model inferred from data from n=5 mice[29]; 2) simulated/actual samples from model inferred from data from n=7 germ-free mice[29]	None	1) 13 taxa (Species); 2) 17 strains (Clostridia)	r^2 (simulated vs. forecasted, mean per taxon) $\approx 0.6 - 0.9$ under various noise levels
PALM[28]	Dynamic Bayesian Network	temporally aligns multi-omics data (taxa, genes, metabolites, environmentals)	Markov assumption; assume fixed number of "parents" for each node	Actual: n=50 patients with inflammatory disease [99]	None	101 taxa (Species); 72 genes; 70 metabolites	MAE (actual vs. forecasted relative abundance per taxon): < 0.004

Continued on next page

Reference	Model	Comments	Limitations	Training Data	Validation Data	Number of Taxa/Samples	Results
Q-net	Recursive Ensemble of Conditional Inference Trees	nonparametric model of n-way dependences; no temporal or other restrictions on abundance dependencies	Applies discretization to relative abundance values; does not directly model measurement noise	Actual: n=58 preterm infants (gut)	Actual: External cohort of n=30 preterm infants (gut)	91 taxa (Class); 398 samples	$R^2 = 0.64$ (actual vs. forecasted relative abundance on out-of-sample prediction of key taxa)

TABLE S3: AUC comparison for classification using the Q-net-induced risk and different sets of additional clinical variables as additional features. See Supplementary Table S10 for definitions of these variables.

**No clinical variables**			**Clinical variable: VD**			**Clinical vars: VD, BW, BHC, EGA**		
week	auc	loc	week	auc	loc	week	auc	loc
26	0.528425	UChicago	26	0.666833	UChicago	26	0.685467	UChicago
27	0.682167	UChicago	27	0.762333	UChicago	27	0.832458	UChicago
28	0.659325	UChicago	28	0.774917	UChicago	28	0.728617	UChicago
29	0.778592	UChicago	29	0.810833	UChicago	29	0.819358	UChicago
30	0.840783	UChicago	30	0.853417	UChicago	30	0.853817	UChicago
31	0.856408	UChicago	31	0.895058	UChicago	31	0.854533	UChicago
32	0.875975	UChicago	32	0.861208	UChicago	32	0.843417	UChicago
33	0.838825	UChicago	33	0.827650	UChicago	33	0.850783	UChicago
34	0.800150	UChicago	34	0.797033	UChicago	34	0.844833	UChicago
35	0.806400	UChicago	35	0.857558	UChicago	35	0.872358	UChicago
26	0.505500	Boston	26	0.500000	Boston	26	0.702854	Boston
27	0.500000	Boston	27	0.519625	Boston	27	0.690875	Boston
28	0.549458	Boston	28	0.532667	Boston	28	0.703979	Boston
29	0.551375	Boston	29	0.584792	Boston	29	0.702646	Boston
30	0.557792	Boston	30	0.626750	Boston	30	0.732667	Boston
31	0.602958	Boston	31	0.651292	Boston	31	0.730167	Boston
32	0.591458	Boston	32	0.651792	Boston	32	0.756625	Boston

TABLE S4: Performance of baseline classification models using only clinical features, and forecasting models using only relative abundances (and not the Q-net-induced risk).

Cohort	Model	Result
UChicago	Random forest (VD)	Median AUC: 0.393
Boston	Random forest (VD, BW, BHC, EGA)	Median AUC: 0.624
UChicago	Dynamic bayesian network (2-stage, all taxa)	Mean R^2 (top taxa): 0.14
UChicago	Dynamic bayesian network (3-stage, all taxa)	Mean R^2 (top taxa): 0.25
UChicago	Dynamic bayesian network (4-stage, all taxa)	Mean R^2 (top taxa): 0.29
UChicago	Dynamic bayesian network (5-stage, all taxa)	Mean R^2 (top taxa): 0.25
UChicago	Dynamic bayesian network (6-stage, all taxa)	Mean R^2 (top taxa): 0.30
Boston	Dynamic bayesian network (2-stage, all taxa)	Mean R^2 (top taxa): 0.21
Boston	Dynamic bayesian network (3-stage, all taxa)	Mean R^2 (top taxa): 0.30
Boston	Dynamic bayesian network (4-stage, all taxa)	Mean R^2 (top taxa): 0.23
Boston	Dynamic bayesian network (5-stage, all taxa)	Mean R^2 (top taxa): 0.29
Boston	Dynamic bayesian network (6-stage, all taxa)	Mean R^2 (top taxa): 0.25

TABLE S5: LOMAR values, appropriate HCG, weeks < 30

	Model	Time	Source	Target	LOMAR (mean)	var	med	95% CI
0	AHCG	28.0	Corio.	Clostridia	86.21	8109.21	85.38	(29.0, 143.43)
1	AHCG	28.0	Corio.	Actino.	8.18	72.98	8.11	(2.75, 13.61)
2	AHCG	29.0	Negativ.	Gammapro.	-7.73	0.00	-7.72	(-7.75, -7.72)
3	AHCG	29.0	MBNT15	Bacteroidia	-4.77	34.75	0.00	(-8.51, -1.02)
4	AHCG	28.0	unclass. Proteo.	Actino.	3.81	31.75	0.00	(0.23, 7.39)
5	AHCG	28.0	Negativ.	Gammapro.	2.85	0.00	2.85	(2.85, 2.86)
6	AHCG	28.0	unclass. Proteo.	Alphapro.	1.73	4.56	0.00	(0.37, 3.08)
7	AHCG	29.0	Nitrospiria	Alphapro.	1.37	1.46	2.33	(0.6, 2.14)
8	AHCG	29.0	MBNT15	Alphapro.	0.70	1.06	0.00	(0.04, 1.35)
9	AHCG	29.0	Clostridia	Clostridia	0.37	0.00	0.37	(0.37, 0.37)
10	AHCG	29.0	Gammapro.	Clostridia	-0.34	0.00	-0.34	(-0.35, -0.34)

Continued on next page

TABLE S5: continued

	Model	Time	Source	Target	LOMAR (mean)	var	med	95% CI
11	AHCG	28.0	unclass. Proteo.	Bacilli_A	0.30	0.10	0.30	(0.1, 0.51)
12	AHCG	28.0	Corio.	BSN033	0.29	0.18	0.00	(0.02, 0.56)
13	AHCG	28.0	unclass. Proteo.	Desulfuro.	0.24	0.09	0.00	(0.05, 0.43)
14	AHCG	29.0	Nitrospiria	Actino.	-0.22	0.11	0.00	(-0.43, -0.01)
15	AHCG	28.0	unclass. Proteo.	UBA10030	0.20	0.09	0.00	(0.01, 0.39)
16	AHCG	28.0	unclass. Proteo.	Ignavi.	0.17	0.05	0.00	(0.04, 0.31)
17	AHCG	28.0	unclass. Proteo.	MBNT15	0.17	0.03	0.16	(0.06, 0.28)
18	AHCG	28.0	Corio.	Methano.	0.13	0.03	0.00	(0.03, 0.24)
19	AHCG	27.0	Gammapro.	Alphapro.	-0.12	0.00	-0.12	(-0.12, -0.12)
20	AHCG	27.0	Gammapro.	unclass. Bact.	-0.11	0.00	-0.11	(-0.11, -0.11)
21	AHCG	28.0	unclass. Proteo.	Desulfobaccia_A	0.09	0.01	0.09	(0.03, 0.16)
22	AHCG	29.0	Syntrophia	MBNT15	0.09	0.01	0.00	(0.02, 0.16)
23	AHCG	28.0	Corio.	Desulfobaccia_A	0.09	0.02	0.00	(0.01, 0.17)
24	AHCG	29.0	Syntrophia	Nitrospiria	0.08	0.01	0.00	(0.02, 0.14)
25	AHCG	29.0	Nitrospiria	Syntrophia	0.07	0.01	0.07	(0.02, 0.12)
26	AHCG	28.0	unclass. Proteo.	Fuso.	0.07	0.01	0.00	(0.0, 0.13)
27	AHCG	28.0	unclass. Proteo.	Thermoleo.	0.06	0.01	0.00	(0.0, 0.12)
28	AHCG	29.0	MBNT15	Nitrospiria	0.05	0.00	0.00	(0.0, 0.09)
29	AHCG	28.0	unclass. Proteo.	Campylo.	0.04	0.00	0.00	(0.0, 0.09)
30	AHCG	28.0	Gammapro.	Negativ.	0.04	0.00	0.04	(0.04, 0.04)
31	AHCG	27.0	Gammapro.	Bacilli	-0.04	0.00	-0.04	(-0.04, -0.04)
32	AHCG	29.0	MBNT15	Anaerolineae	0.04	0.00	0.00	(0.01, 0.07)
33	AHCG	27.0	Gammapro.	Bacteroidia	-0.04	0.00	-0.04	(-0.04, -0.04)
34	AHCG	29.0	Syntrophia	Campylo.	0.04	0.00	0.04	(0.01, 0.06)
35	AHCG	27.0	Gammapro.	Vicinami.	-0.03	0.00	-0.03	(-0.03, -0.03)
36	AHCG	28.0	Alphapro.	Bacilli_A	0.03	0.00	0.03	(0.01, 0.05)
37	AHCG	29.0	Nitrospiria	Ellin6529	0.03	0.00	0.03	(0.01, 0.04)
38	AHCG	29.0	Nitrospiria	Pacei._A	0.02	0.00	0.02	(0.02, 0.02)
39	AHCG	29.0	Syntrophia	Desulfobaccia_A	0.02	0.00	0.02	(0.01, 0.03)
40	AHCG	29.0	Alphapro.	Vicinami.	0.02	0.00	0.02	(0.01, 0.03)
41	AHCG	28.0	Actino.	Vicinami.	0.02	0.00	0.00	(0.0, 0.03)
42	AHCG	27.0	Gammapro.	Clostridia	-0.01	0.00	-0.01	(-0.02, -0.01)
43	AHCG	29.0	Syntrophia	Gemmatimo.	0.01	0.00	0.00	(0.0, 0.02)
44	AHCG	27.0	Gammapro.	MBNT15	-0.01	0.00	-0.01	(-0.01, -0.01)
45	AHCG	29.0	Verruco.	Myxococcia	0.01	0.00	0.00	(0.0, 0.02)
46	AHCG	29.0	MBNT15	Fimbrii.	0.01	0.00	0.00	(0.0, 0.02)
47	AHCG	29.0	Syntrophia	Pacei._A	0.01	0.00	0.00	(0.0, 0.02)
48	AHCG	29.0	Nitrospiria	Oxyphoto.	0.01	0.00	0.00	(0.0, 0.02)
49	AHCG	29.0	Nitrospiria	Thermoleo.	0.01	0.00	0.00	(0.0, 0.02)
50	AHCG	27.0	Gammapro.	Anaerolineae	-0.01	0.00	-0.01	(-0.01, -0.01)
51	AHCG	28.0	unclass. Proteo.	Pacei._A	0.01	0.00	0.00	(0.0, 0.01)
52	AHCG	29.0	MBNT15	Oxyphoto.	0.01	0.00	0.00	(0.0, 0.01)
53	AHCG	29.0	MBNT15	Polyangia	0.01	0.00	0.00	(0.0, 0.01)
54	AHCG	29.0	Verruco.	Desulfobaccia_A	0.01	0.00	0.01	(0.0, 0.01)
55	AHCG	29.0	Alphapro.	Doudna.	0.01	0.00	0.00	(0.0, 0.01)
56	AHCG	27.0	Gammapro.	Blasto.	-0.01	0.00	-0.01	(-0.01, -0.01)
57	AHCG	29.0	Nitrospiria	Planctomycetes	0.01	0.00	0.00	(0.0, 0.01)
58	AHCG	28.0	Actino.	Bacilli_A	0.01	0.00	0.00	(0.0, 0.01)
59	AHCG	27.0	Gammapro.	Ignavi.	-0.01	0.00	-0.01	(-0.01, -0.01)
60	AHCG	29.0	Verruco.	Acido.	0.01	0.00	0.01	(0.0, 0.01)
61	AHCG	28.0	Alphapro.	Desulfobaccia_A	0.01	0.00	0.00	(0.0, 0.01)
62	AHCG	29.0	MBNT15	Planctomycetes	0.01	0.00	0.00	(0.0, 0.01)
63	AHCG	28.0	Actino.	unclass. Desulfobact	0.01	0.00	0.01	(0.0, 0.01)
64	AHCG	28.0	Alphapro.	Holophagae	0.01	0.00	0.00	(0.0, 0.01)
65	AHCG	29.0	Negativ.	Aminicenantia	0.00	0.00	0.00	(0.0, 0.01)

Continued on next page

TABLE S5: continued

	Model	Time	Source	Target	LOMAR (mean)	var	med	95% CI
66	AHCG	28.0	Actino.	Corio.	0.00	0.00	0.00	(0.0, 0.01)
67	AHCG	29.0	Alphapro.	MBNT15	0.00	0.00	0.00	(0.0, 0.01)
68	AHCG	28.0	Actino.	unclass. Proteo.	0.00	0.00	0.00	(0.0, 0.01)
69	AHCG	27.0	Gammapro.	Myxococcia	-0.00	0.00	-0.00	(-0.0, -0.0)
70	AHCG	29.0	Negativ.	Methano.	0.00	0.00	0.00	(0.0, 0.0)
71	AHCG	27.0	Gammapro.	Campylo.	-0.00	0.00	-0.00	(-0.0, -0.0)
72	AHCG	27.0	Gammapro.	unclass. Proteo.	-0.00	0.00	-0.00	(-0.0, -0.0)
73	AHCG	28.0	Alphapro.	Desulfo.	0.00	0.00	0.00	(0.0, 0.01)
74	AHCG	28.0	Actino.	Desulfobaccia_A	0.00	0.00	0.00	(0.0, 0.0)
75	AHCG	29.0	Verruco.	Microgenomatia	0.00	0.00	0.00	(0.0, 0.0)
76	AHCG	29.0	Alphapro.	Desulfobaccia_A	0.00	0.00	0.00	(0.0, 0.0)
77	AHCG	29.0	Alphapro.	Pacei._A	0.00	0.00	0.00	(0.0, 0.0)
78	AHCG	29.0	Verruco.	Cyano.	0.00	0.00	0.00	(0.0, 0.0)
79	AHCG	28.0	Alphapro.	Pacei._A	0.00	0.00	0.00	(0.0, 0.0)
80	AHCG	28.0	Clostridia	Desulfovi.	0.00	0.00	0.00	(0.0, 0.0)
81	AHCG	28.0	Clostridia	Bacilli_A	0.00	0.00	0.00	(0.0, 0.0)
82	AHCG	28.0	Clostridia	unclass. Proteo.	0.00	0.00	0.00	(0.0, 0.0)
83	AHCG	29.0	Alphapro.	Gemmatimo.	0.00	0.00	0.00	(0.0, 0.0)
84	AHCG	27.0	Gammapro.	Thermodesulfovi.	-0.00	0.00	-0.00	(-0.0, -0.0)
85	AHCG	28.0	Actino.	Pacei._A	0.00	0.00	0.00	(0.0, 0.0)
86	AHCG	28.0	Actino.	Desulfobulbia	0.00	0.00	0.00	(0.0, 0.0)
87	AHCG	29.0	unclass. Bact.	Acidimicrobiia	0.00	0.00	0.00	(0.0, 0.0)
88	AHCG	28.0	Clostridia	Corio.	0.00	0.00	0.00	(0.0, 0.0)
89	AHCG	28.0	Bacteroidia	Myxococcia	0.00	0.00	0.00	(0.0, 0.0)
90	AHCG	28.0	Clostridia	Thermoleo.	0.00	0.00	0.00	(0.0, 0.0)
91	AHCG	28.0	Bacteroidia	Desulfobaccia_A	0.00	0.00	0.00	(0.0, 0.0)
92	AHCG	29.0	unclass. Bact.	Fuso.	0.00	0.00	0.00	(0.0, 0.0)
93	AHCG	28.0	Bacteroidia	Thermodesulfovi.	0.00	0.00	0.00	(0.0, 0.0)
94	AHCG	29.0	unclass. Bact.	Methylomirabilia	0.00	0.00	0.00	(0.0, 0.0)
95	AHCG	28.0	Clostridia	Cyano.	0.00	0.00	0.00	(0.0, 0.0)
96	AHCG	29.0	unclass. Bact.	Desulfobulbia	0.00	0.00	0.00	(0.0, 0.0)
97	AHCG	28.0	Bacteroidia	Pacei._A	0.00	0.00	0.00	(0.0, 0.0)

TABLE S6: LOMAR values, appropriate HCG, weeks ≥ 30

	Model	Time	Source	Target	LOMAR (mean)	var	med	95% CI
0	AHCG	30.0	Actino.	Gammapro.	-22.68	0.00	-22.69	(-22.71, -22.65)
1	AHCG	31.0	Corio.	Actino.	15.86	0.07	15.89	(15.69, 16.02)
2	AHCG	31.0	Corio.	Clostridia	11.43	0.00	11.42	(11.39, 11.47)
3	AHCG	32.0	Bacilli	Gammapro.	4.30	0.36	4.13	(3.92, 4.68)
4	AHCG	32.0	Vicinami.	Actino.	-2.01	6.15	0.00	(-3.58, -0.43)
5	AHCG	32.0	Corio.	Actino.	1.86	0.00	1.86	(1.85, 1.87)
6	AHCG	30.0	Actino.	Clostridia	1.36	0.18	1.49	(1.09, 1.64)
7	AHCG	32.0	Gammapro.	Gammapro.	1.34	0.01	1.31	(1.27, 1.41)
8	AHCG	30.0	Negativ.	Gammapro.	-1.18	0.51	-1.57	(-1.63, -0.73)
9	AHCG	32.0	Desulfuro.	Alphapro.	0.82	1.45	0.00	(0.05, 1.58)
10	AHCG	32.0	Desulfuro.	unclass. Bacteroidot	0.65	0.47	0.65	(0.22, 1.09)
11	AHCG	32.0	Desulfuro.	Thermoleo.	0.55	0.45	0.00	(0.12, 0.97)
12	AHCG	30.0	Corio.	Alphapro.	0.45	0.00	0.45	(0.44, 0.46)
13	AHCG	32.0	Desulfuro.	Bacteriov.	0.44	0.42	0.00	(0.03, 0.85)
14	AHCG	32.0	Bacteroidia	Gammapro.	-0.43	0.00	-0.45	(-0.46, -0.41)
15	AHCG	32.0	Actino.	Clostridia	0.35	0.00	0.36	(0.34, 0.37)
16	AHCG	32.0	Bacilli	unclass. Bact.	-0.33	0.00	-0.34	(-0.34, -0.33)
17	AHCG	31.0	unclass. Proteo.	Bacteroidia	-0.29	0.00	-0.30	(-0.31, -0.28)

Continued on next page

TABLE S6: continued

	Model	Time	Source	Target	LOMAR (mean)	var	med	95% CI
18	AHCG	31.0	Actino.	unclass. Bact.	0.27	0.00	0.27	(0.27, 0.27)
19	AHCG	31.0	Clostridia	Bacteroidia	0.27	0.00	0.27	(0.26, 0.27)
20	AHCG	32.0	Corio.	Clostridia	-0.25	0.00	-0.26	(-0.27, -0.23)
21	AHCG	32.0	Fuso.	Polyangia	0.22	0.10	0.00	(0.01, 0.42)
22	AHCG	32.0	Oxyphoto.	Bacilli_A	0.13	0.01	0.19	(0.07, 0.19)
23	AHCG	30.0	Corio.	Bacilli_A	0.11	0.00	0.11	(0.11, 0.11)
24	AHCG	30.0	Bacteroidia	Bacteroidia	0.11	0.00	0.11	(0.08, 0.13)
25	AHCG	32.0	Clostridia	Clostridia	0.10	0.01	0.17	(0.04, 0.16)
26	AHCG	32.0	Fuso.	Nitrospiria	0.09	0.01	0.08	(0.03, 0.14)
27	AHCG	32.0	Vicinami.	Fuso.	0.09	0.00	0.13	(0.05, 0.13)
28	AHCG	32.0	unclass. Bact.	Bacilli	0.07	0.00	0.11	(0.05, 0.1)
29	AHCG	32.0	Desulfuro.	Bacilli_A	0.07	0.00	0.09	(0.05, 0.09)
30	AHCG	32.0	Desulfuro.	Fuso.	0.07	0.01	0.00	(0.0, 0.13)
31	AHCG	30.0	Actino.	Corio.	0.06	0.00	0.06	(0.06, 0.06)
32	AHCG	31.0	Bacilli	Actino.	0.04	0.00	0.06	(0.02, 0.06)
33	AHCG	32.0	Desulfuro.	MBNT15	0.04	0.00	0.00	(0.0, 0.07)
34	AHCG	30.0	Corio.	Vicinami.	0.04	0.00	0.04	(0.04, 0.04)
35	AHCG	30.0	Bacilli	Clostridia	0.04	0.00	0.03	(0.03, 0.04)
36	AHCG	32.0	Vicinami.	Desulfuro.	0.04	0.00	0.04	(0.01, 0.06)
37	AHCG	30.0	Corio.	Nitrospiria	0.03	0.00	0.03	(0.03, 0.03)
38	AHCG	31.0	Gammapro.	Actino.	0.03	0.00	0.04	(0.01, 0.05)
39	AHCG	30.0	Negativ.	Clostridia	0.03	0.00	0.05	(0.01, 0.05)
40	AHCG	32.0	Bacteroidia	unclass. Proteo.	-0.03	0.00	-0.03	(-0.03, -0.03)
41	AHCG	32.0	Fuso.	Desulfuro.	0.03	0.00	0.03	(0.01, 0.05)
42	AHCG	31.0	Negativ.	Actino.	0.03	0.00	0.02	(0.01, 0.04)
43	AHCG	30.0	Corio.	Verruco.	0.02	0.00	0.02	(0.02, 0.03)
44	AHCG	32.0	Bacteroidia	Clostridia	0.02	0.00	0.00	(0.01, 0.04)
45	AHCG	30.0	Corio.	Ellin6529	0.02	0.00	0.02	(0.02, 0.02)
46	AHCG	32.0	Gammapro.	Clostridia	0.02	0.00	0.02	(0.01, 0.03)
47	AHCG	32.0	Bacilli	Corio.	0.02	0.00	0.02	(0.02, 0.02)
48	AHCG	32.0	Vicinami.	Bacilli_A	0.02	0.00	0.00	(0.0, 0.04)
49	AHCG	31.0	Verruco.	Methano.	0.02	0.00	0.02	(0.02, 0.02)
50	AHCG	30.0	Bacteroidia	Clostridia	0.01	0.00	0.00	(0.0, 0.02)
51	AHCG	30.0	Corio.	Actino.	0.01	0.00	0.01	(0.01, 0.02)
52	AHCG	32.0	Actino.	Bacilli	-0.01	0.00	-0.02	(-0.02, -0.01)
53	AHCG	32.0	Fuso.	Vicinami.	0.01	0.00	0.02	(0.01, 0.02)
54	AHCG	30.0	Corio.	Blasto.	0.01	0.00	0.01	(0.01, 0.01)
55	AHCG	30.0	unclass. Bact.	Actino.	0.01	0.00	0.01	(0.01, 0.01)
56	AHCG	31.0	Corio.	Desulfovi.	0.01	0.00	0.01	(0.01, 0.01)
57	AHCG	31.0	Clostridia	Corio.	0.01	0.00	0.01	(0.01, 0.01)
58	AHCG	30.0	Corio.	Oxyphoto.	0.01	0.00	0.01	(0.01, 0.01)
59	AHCG	32.0	Actino.	Corio.	0.00	0.00	0.00	(0.0, 0.0)
60	AHCG	31.0	Actino.	Corio.	-0.00	0.00	-0.00	(-0.0, -0.0)
61	AHCG	32.0	unclass. Bact.	Deferri.	0.00	0.00	0.00	(0.0, 0.0)
62	AHCG	32.0	unclass. Bact.	Corio.	0.00	0.00	0.00	(0.0, 0.0)
63	AHCG	31.0	Negativ.	Clostridia	0.00	0.00	0.00	(0.0, 0.0)
64	AHCG	32.0	Negativ.	Deferri.	0.00	0.00	0.00	(0.0, 0.0)
65	AHCG	31.0	Clostridia	Oxyphoto.	0.00	0.00	0.00	(0.0, 0.0)
66	AHCG	31.0	Actino.	Oxyphoto.	0.00	0.00	0.00	(0.0, 0.0)
67	AHCG	31.0	unclass. Proteo.	Fuso.	0.00	0.00	0.00	(0.0, 0.0)
68	AHCG	31.0	Bacteroidia	Nitrospiria	0.00	0.00	0.00	(0.0, 0.0)
69	AHCG	32.0	Gammapro.	Corio.	0.00	0.00	0.00	(0.0, 0.0)
70	AHCG	31.0	unclass. Bact.	Nitrospiria	0.00	0.00	0.00	(0.0, 0.0)
71	AHCG	31.0	Gammapro.	Oxyphoto.	0.00	0.00	0.00	(0.0, 0.0)
72	AHCG	31.0	unclass. Bact.	Fuso.	0.00	0.00	0.00	(0.0, 0.0)
73	AHCG	32.0	Gammapro.	Deferri.	0.00	0.00	0.00	(0.0, 0.0)

Continued on next page

TABLE S6: continued

	Model	Time	Source	Target	LOMAR (mean)	var	med	95% CI
74	AHCG	31.0	Gammapro.	Nitrospiria	0.00	0.00	0.00	(0.0, 0.0)

TABLE S7: LOMAR values, suboptimal HCG, weeks < 30

	Model	Time	Source	Target	LOMAR (mean)	var	med	95% CI
0	SHCG	29.0	Verruco.	Gammapro.	-251.07	32.29	-255.32	(-254.68, -247.46)
1	SHCG	27.0	Corio.	Clostridia	15.11	348.84	0.00	(3.25, 26.98)
2	SHCG	29.0	Bacilli	Gammapro.	12.08	222.98	0.00	(2.6, 21.57)
3	SHCG	28.0	Corio.	Verruco.	8.31	0.02	8.30	(8.23, 8.39)
4	SHCG	29.0	Actino.	Actino.	8.17	0.00	8.16	(8.13, 8.2)
5	SHCG	27.0	Deferri.	Negativ.	5.63	0.07	5.55	(5.46, 5.8)
6	SHCG	27.0	Corio.	Fuso.	4.71	24.16	4.64	(1.58, 7.83)
7	SHCG	28.0	Corio.	Vicinami.	3.18	11.06	3.14	(1.07, 5.3)
8	SHCG	28.0	Corio.	unclass. Actino.	2.76	5.93	4.71	(1.21, 4.3)
9	SHCG	27.0	Actino.	unclass. Bact.	-2.36	12.16	0.00	(-4.58, -0.15)
10	SHCG	28.0	Corio.	Bacteroidia	-2.33	5.93	-2.26	(-3.88, -0.78)
11	SHCG	27.0	Verruco.	Clostridia	2.24	3.94	3.73	(0.98, 3.5)
12	SHCG	28.0	Corio.	Nitrosos.	1.97	5.93	0.00	(0.42, 3.52)
13	SHCG	27.0	Corio.	Verruco.	1.95	0.00	1.95	(1.94, 1.96)
14	SHCG	28.0	Corio.	Anaerolineae	0.89	1.22	0.00	(0.19, 1.59)
15	SHCG	28.0	Corio.	Nitrospiria	0.67	0.99	0.00	(0.04, 1.3)
16	SHCG	28.0	Corio.	MBNT15	0.66	0.95	0.00	(0.04, 1.28)
17	SHCG	28.0	Corio.	Desulfobaccia_A	0.64	0.00	0.64	(0.64, 0.64)
18	SHCG	27.0	Corio.	Oxyphoto.	0.53	0.22	0.90	(0.23, 0.82)
19	SHCG	27.0	Verruco.	Fuso.	0.49	0.26	0.48	(0.16, 0.81)
20	SHCG	29.0	Actino.	Bacilli	0.43	0.00	0.43	(0.43, 0.43)
21	SHCG	28.0	Corio.	Syntrophia	0.41	0.36	0.00	(0.02, 0.79)
22	SHCG	27.0	Bacilli	Actino.	0.38	0.00	0.38	(0.38, 0.38)
23	SHCG	27.0	Clostridia	unclass. Bact.	-0.38	0.31	0.00	(-0.73, -0.02)
24	SHCG	28.0	Corio.	Pacei._A	0.37	0.03	0.44	(0.26, 0.48)
25	SHCG	27.0	Clostridia	Actino.	0.29	0.00	0.29	(0.28, 0.29)
26	SHCG	28.0	Corio.	Myxococcia	0.27	0.08	0.27	(0.09, 0.46)
27	SHCG	28.0	Corio.	Desulfo.	0.24	0.08	0.00	(0.05, 0.42)
28	SHCG	28.0	Corio.	Desulfobulbia	0.20	0.06	0.00	(0.04, 0.36)
29	SHCG	28.0	Corio.	Acido.	0.19	0.06	0.00	(0.04, 0.34)
30	SHCG	28.0	Corio.	Gemmatimo.	0.17	0.06	0.00	(0.01, 0.33)
31	SHCG	29.0	Gammapro.	Bacilli	0.15	0.00	0.15	(0.15, 0.15)
32	SHCG	29.0	unclass. Bact.	unclass. Bact.	0.15	0.02	0.14	(0.05, 0.24)
33	SHCG	27.0	Clostridia	Bacteroidia	0.15	0.00	0.15	(0.14, 0.15)
34	SHCG	28.0	Verruco.	Alphapro.	0.13	0.04	0.00	(0.01, 0.25)
35	SHCG	27.0	Gammapro.	unclass. Bact.	-0.13	0.03	0.00	(-0.25, -0.01)
36	SHCG	27.0	Actino.	Bacilli	0.10	0.00	0.10	(0.1, 0.1)
37	SHCG	29.0	Actino.	Clostridia	0.08	0.00	0.08	(0.08, 0.08)
38	SHCG	29.0	unclass. Bact.	Gammapro.	-0.08	0.01	0.00	(-0.14, -0.02)
39	SHCG	29.0	Gammapro.	unclass. Bact.	-0.06	0.01	-0.04	(-0.12, -0.0)
40	SHCG	28.0	Verruco.	Bacteroidia	-0.04	0.00	-0.04	(-0.07, -0.01)
41	SHCG	28.0	Bacteroidia	Alphapro.	0.04	0.00	0.00	(0.01, 0.08)
42	SHCG	27.0	Verruco.	Oxyphoto.	0.04	0.00	0.00	(0.01, 0.07)
43	SHCG	28.0	Verruco.	Nitrospiria	0.03	0.00	0.04	(0.01, 0.04)
44	SHCG	27.0	Verruco.	Corio.	0.02	0.00	0.02	(0.02, 0.02)
45	SHCG	28.0	Verruco.	MBNT15	0.02	0.00	0.02	(0.01, 0.03)
46	SHCG	28.0	Bacteroidia	Chlamydiia	0.02	0.00	0.02	(0.01, 0.02)
47	SHCG	28.0	Verruco.	Anaerolineae	0.01	0.00	0.00	(0.0, 0.03)
48	SHCG	28.0	Bacteroidia	unclass. Desulfobact	0.01	0.00	0.01	(0.0, 0.02)
49	SHCG	29.0	Actino.	Alphapro.	0.01	0.00	0.01	(0.01, 0.01)

Continued on next page

TABLE S7: continued

	Model	Time	Source	Target	LOMAR (mean)	var	med	95% CI
50	SHCG	28.0	Verruco.	Desulfuro.	0.01	0.00	0.00	(0.0, 0.02)
51	SHCG	28.0	Bacilli	Negativ.	-0.01	0.00	-0.01	(-0.01, -0.01)
52	SHCG	28.0	Bacteroidia	Vicinami.	0.01	0.00	0.00	(0.0, 0.02)
53	SHCG	28.0	Bacteroidia	Nitrosos.	0.01	0.00	0.00	(0.0, 0.02)
54	SHCG	28.0	Verruco.	Campylo.	0.01	0.00	0.00	(0.0, 0.02)
55	SHCG	28.0	Verruco.	Desulfobaccia_A	0.01	0.00	0.01	(0.0, 0.01)
56	SHCG	28.0	Verruco.	Syntrophia	0.01	0.00	0.00	(0.0, 0.02)
57	SHCG	28.0	Verruco.	unclass. Chloroflexo	0.01	0.00	0.01	(0.0, 0.01)
58	SHCG	28.0	Bacteroidia	Ignavi.	0.01	0.00	0.01	(0.0, 0.01)
59	SHCG	28.0	Bacteroidia	Desulfovi.	0.01	0.00	0.00	(0.0, 0.01)
60	SHCG	27.0	Bacteroidia	Clostridia	-0.01	0.00	0.00	(-0.01, -0.0)
61	SHCG	28.0	Verruco.	Pacei._A	0.00	0.00	0.00	(0.0, 0.01)
62	SHCG	28.0	Verruco.	Acido.	0.00	0.00	0.00	(0.0, 0.01)
63	SHCG	28.0	Bacteroidia	Desulfuro.	0.00	0.00	0.00	(0.0, 0.01)
64	SHCG	28.0	Bacteroidia	Fuso.	0.00	0.00	0.01	(0.0, 0.0)
65	SHCG	29.0	Actino.	Blasto.	0.00	0.00	0.00	(0.0, 0.0)
66	SHCG	28.0	Bacteroidia	Campylo.	0.00	0.00	0.00	(0.0, 0.0)
67	SHCG	28.0	Verruco.	Corio.	0.00	0.00	0.00	(0.0, 0.0)
68	SHCG	28.0	Bacteroidia	Syntrophia	0.00	0.00	0.00	(0.0, 0.0)
69	SHCG	27.0	Actino.	Clostridia	-0.00	0.00	-0.00	(-0.0, -0.0)
70	SHCG	28.0	Bacteroidia	Holophagae	0.00	0.00	0.00	(0.0, 0.0)
71	SHCG	28.0	Bacteroidia	Pacei._A	0.00	0.00	0.00	(0.0, 0.0)
72	SHCG	28.0	Bacteroidia	Polyangia	0.00	0.00	0.00	(0.0, 0.0)
73	SHCG	29.0	Actino.	unclass. Proteo.	0.00	0.00	0.00	(0.0, 0.0)
74	SHCG	28.0	Verruco.	Microgenomatia	0.00	0.00	0.00	(0.0, 0.0)
75	SHCG	28.0	Bacteroidia	Gemmatimo.	0.00	0.00	0.00	(0.0, 0.0)
76	SHCG	28.0	Bacteroidia	Desulfobaccia_A	0.00	0.00	0.00	(0.0, 0.0)
77	SHCG	28.0	Bacteroidia	Planctomycetes	0.00	0.00	0.00	(0.0, 0.0)
78	SHCG	28.0	Bacteroidia	Desulfo.	0.00	0.00	0.00	(0.0, 0.0)
79	SHCG	28.0	Bacteroidia	Desulfobulbia	0.00	0.00	0.00	(0.0, 0.0)
80	SHCG	27.0	unclass. Bact.	Sacchari.	0.00	0.00	0.00	(0.0, 0.0)
81	SHCG	27.0	Gammapro.	Bacilli	0.00	0.00	0.00	(0.0, 0.0)
82	SHCG	28.0	Bacteroidia	Microgenomatia	0.00	0.00	0.00	(0.0, 0.0)

TABLE S8: LOMAR values, suboptimal HCG, weeks ≥ 31

	Model	Time	Source	Target	LOMAR (mean)	var	med	95% CI
0	SHCG	30.0	Verruco.	unclass. Bact.	257.64	6588.12	280.77	(206.07, 309.21)
1	SHCG	30.0	MBNT15	Gammapro.	-98.77	5322.03	-147.29	(-145.12, -52.42)
2	SHCG	30.0	Verruco.	Bacteroidia	41.42	0.40	41.41	(41.01, 41.82)
3	SHCG	30.0	Deferri.	Negativ.	13.47	0.04	13.42	(13.35, 13.59)
4	SHCG	30.0	Alphapro.	Gammapro.	-5.24	59.85	0.00	(-10.15, -0.32)
5	SHCG	31.0	unclass. Bact.	Gammapro.	-4.70	2.19	-5.12	(-5.64, -3.76)
6	SHCG	30.0	Bacteroidia	Bacilli	4.21	4.15	4.61	(2.91, 5.5)
7	SHCG	30.0	Verruco.	Negativ.	2.95	0.03	2.92	(2.85, 3.06)
8	SHCG	32.0	Bacilli	Gammapro.	-2.42	5.45	-2.43	(-3.9, -0.94)
9	SHCG	31.0	Nitrospiria	Alphapro.	0.96	1.42	0.00	(0.21, 1.72)
10	SHCG	30.0	Bacilli	unclass. Bact.	-0.93	0.25	-1.07	(-1.25, -0.61)
11	SHCG	31.0	Ignavi.	Desulfobaccia_A	0.83	0.00	0.83	(0.83, 0.83)
12	SHCG	31.0	Actino.	unclass. Bact.	0.81	0.06	0.70	(0.66, 0.96)
13	SHCG	30.0	Verruco.	Corio.	0.81	0.00	0.81	(0.8, 0.81)
14	SHCG	32.0	Negativ.	Gammapro.	-0.73	0.36	-0.97	(-1.11, -0.34)
15	SHCG	32.0	Negativ.	unclass. Bact.	0.52	0.54	0.00	(0.05, 0.99)
16	SHCG	32.0	unclass. Bact.	Gammapro.	0.33	0.26	0.71	(0.0, 0.66)
17	SHCG	32.0	Corio.	Clostridia	0.33	0.00	0.32	(0.31, 0.35)

Continued on next page

TABLE S8: continued

	Model	Time	Source	Target	LOMAR (mean)	var	med	95% CI
18	SHCG	31.0	Ignavi.	Desulfobulbia	0.28	0.17	0.00	(0.02, 0.54)
19	SHCG	30.0	Actino.	Bacilli	-0.26	0.01	-0.29	(-0.33, -0.2)
20	SHCG	31.0	Syntrophia	Gemmatimo.	0.26	0.15	0.00	(0.02, 0.5)
21	SHCG	32.0	Clostridia	Actino.	-0.22	0.00	-0.22	(-0.23, -0.22)
22	SHCG	31.0	Ignavi.	Verruco.	0.20	0.09	0.00	(0.01, 0.39)
23	SHCG	31.0	Blasto.	Nitrospiria	0.20	0.04	0.20	(0.07, 0.34)
24	SHCG	30.0	MBNT15	Nitrosos.	0.20	0.04	0.20	(0.07, 0.33)
25	SHCG	32.0	unclass. Bact.	Bacilli	0.19	0.00	0.20	(0.16, 0.22)
26	SHCG	31.0	Gammapro.	Gammapro.	0.19	0.00	0.20	(0.15, 0.22)
27	SHCG	30.0	Gammapro.	Gammapro.	0.18	0.07	0.00	(0.01, 0.35)
28	SHCG	32.0	Actino.	Clostridia	0.15	0.01	0.20	(0.11, 0.2)
29	SHCG	32.0	unclass. Proteo.	unclass. Proteo.	0.13	0.00	0.13	(0.13, 0.13)
30	SHCG	30.0	Clostridia	Bacilli	-0.10	0.00	-0.11	(-0.13, -0.08)
31	SHCG	30.0	unclass. Proteo.	unclass. Proteo.	0.10	0.00	0.10	(0.1, 0.1)
32	SHCG	31.0	Actino.	Actino.	0.09	0.00	0.09	(0.07, 0.11)
33	SHCG	31.0	Nitrospiria	Verruco.	0.08	0.01	0.00	(0.0, 0.16)
34	SHCG	30.0	Gammapro.	unclass. Proteo.	-0.07	0.01	-0.07	(-0.12, -0.02)
35	SHCG	31.0	Blasto.	Campylo.	0.06	0.00	0.00	(0.01, 0.1)
36	SHCG	31.0	Gammapro.	Negativ.	0.05	0.00	0.06	(0.03, 0.07)
37	SHCG	31.0	unclass. Bact.	Actino.	0.05	0.00	0.05	(0.05, 0.05)
38	SHCG	31.0	Clostridia	Alicyclobacillia	0.04	0.00	0.04	(0.04, 0.04)
39	SHCG	32.0	Vicinami.	Vicinami.	0.04	0.00	0.00	(0.0, 0.07)
40	SHCG	30.0	Bacilli	Actino.	0.04	0.00	0.04	(0.02, 0.06)
41	SHCG	32.0	Gammapro.	Gammapro.	0.04	0.00	0.00	(0.01, 0.06)
42	SHCG	30.0	MBNT15	Anaerolineae	0.03	0.00	0.00	(0.01, 0.06)
43	SHCG	30.0	MBNT15	Nitrospiria	0.03	0.00	0.00	(0.0, 0.06)
44	SHCG	31.0	MBNT15	Corio.	-0.03	0.00	-0.03	(-0.03, -0.03)
45	SHCG	30.0	Verruco.	Desulfovi.	0.03	0.00	0.00	(0.01, 0.05)
46	SHCG	30.0	MBNT15	Syntrophia	0.03	0.00	0.03	(0.01, 0.04)
47	SHCG	30.0	Verruco.	Methano.	0.03	0.00	0.00	(0.01, 0.04)
48	SHCG	30.0	MBNT15	Desulfuro.	0.02	0.00	0.00	(0.0, 0.05)
49	SHCG	31.0	Syntrophia	Blasto.	0.02	0.00	0.00	(0.0, 0.05)
50	SHCG	32.0	Vicinami.	MBNT15	0.02	0.00	0.04	(0.01, 0.03)
51	SHCG	32.0	Vicinami.	Ignavi.	0.02	0.00	0.03	(0.01, 0.03)
52	SHCG	31.0	Nitrospiria	Desulfuro.	0.02	0.00	0.00	(0.0, 0.04)
53	SHCG	32.0	Gammapro.	Bacilli	-0.02	0.00	-0.01	(-0.03, -0.0)
54	SHCG	31.0	Ignavi.	unclass. Actino.	0.02	0.00	0.00	(0.0, 0.03)
55	SHCG	32.0	Alphapro.	Vicinami.	0.02	0.00	0.03	(0.01, 0.03)
56	SHCG	30.0	Alphapro.	Vicinami.	0.02	0.00	0.00	(0.0, 0.03)
57	SHCG	32.0	Vicinami.	Campylo.	0.02	0.00	0.03	(0.01, 0.02)
58	SHCG	32.0	Vicinami.	Anaerolineae	0.01	0.00	0.01	(0.0, 0.02)
59	SHCG	30.0	MBNT15	Campylo.	0.01	0.00	0.00	(0.0, 0.02)
60	SHCG	30.0	MBNT15	Myxococcia	0.01	0.00	0.00	(0.0, 0.02)
61	SHCG	30.0	Gammapro.	Negativ.	0.01	0.00	0.01	(0.01, 0.01)
62	SHCG	30.0	Gammapro.	Bacilli	-0.01	0.00	0.00	(-0.02, -0.0)
63	SHCG	30.0	MBNT15	Fuso.	0.01	0.00	0.00	(0.0, 0.02)
64	SHCG	30.0	Bacilli	Gammapro.	-0.01	0.00	0.00	(-0.02, -0.0)
65	SHCG	30.0	MBNT15	Polyangia	0.01	0.00	0.00	(0.0, 0.01)
66	SHCG	30.0	MBNT15	unclass. Actino.	0.01	0.00	0.00	(0.0, 0.01)
67	SHCG	30.0	Corio.	Verruco.	0.01	0.00	0.01	(0.01, 0.01)
68	SHCG	32.0	Vicinami.	Fuso.	0.01	0.00	0.00	(0.0, 0.01)
69	SHCG	32.0	unclass. Proteo.	Clostridia	0.01	0.00	0.00	(0.0, 0.01)
70	SHCG	30.0	Alphapro.	Nitrospiria	0.00	0.00	0.00	(0.0, 0.01)
71	SHCG	30.0	Alphapro.	Ignavi.	0.00	0.00	0.00	(0.0, 0.01)
72	SHCG	32.0	Negativ.	Thermoanaero.	0.00	0.00	0.00	(0.0, 0.0)
73	SHCG	32.0	Alphapro.	MBNT15	0.00	0.00	0.00	(0.0, 0.01)

Continued on next page

TABLE S8: continued

	Model	Time	Source	Target	LOMAR (mean)	var	med	95% CI
74	SHCG	30.0	Alphapro.	Anaerolineae	0.00	0.00	0.00	(0.0, 0.01)
75	SHCG	30.0	Alphapro.	Desulfuro.	0.00	0.00	0.00	(0.0, 0.01)
76	SHCG	32.0	Alphapro.	Anaerolineae	0.00	0.00	0.00	(0.0, 0.01)
77	SHCG	30.0	Corio.	Desulfovi.	0.00	0.00	0.00	(0.0, 0.01)
78	SHCG	32.0	Alphapro.	Campylo.	0.00	0.00	0.00	(0.0, 0.0)
79	SHCG	32.0	Alphapro.	Ignavi.	0.00	0.00	0.00	(0.0, 0.0)
80	SHCG	32.0	Alphapro.	Fuso.	0.00	0.00	0.00	(0.0, 0.0)
81	SHCG	30.0	Alphapro.	Myxococcia	0.00	0.00	0.00	(0.0, 0.0)
82	SHCG	31.0	unclass. Proteo.	Desulfobaccia_A	0.00	0.00	0.00	(0.0, 0.0)
83	SHCG	30.0	Alphapro.	Verruco.	-0.00	0.00	-0.00	(-0.0, -0.0)
84	SHCG	30.0	Alphapro.	Desulfo.	0.00	0.00	0.00	(0.0, 0.0)
85	SHCG	30.0	Bacteroidia	Thermodesulfovi.	0.00	0.00	0.00	(0.0, 0.0)
86	SHCG	32.0	Bacilli	Campylo.	0.00	0.00	0.00	(0.0, 0.0)
87	SHCG	32.0	Bacilli	Ignavi.	0.00	0.00	0.00	(0.0, 0.0)
88	SHCG	32.0	Actino.	Verruco.	0.00	0.00	0.00	(0.0, 0.0)
89	SHCG	30.0	Alphapro.	Fuso.	0.00	0.00	0.00	(0.0, 0.0)
90	SHCG	30.0	Alphapro.	Planctomycetes	0.00	0.00	0.00	(0.0, 0.0)
91	SHCG	32.0	Bacilli	Anaerolineae	0.00	0.00	0.00	(0.0, 0.0)
92	SHCG	32.0	Vicinami.	Oxyphoto.	0.00	0.00	0.00	(0.0, 0.0)
93	SHCG	30.0	Alphapro.	unclass. Actino.	0.00	0.00	0.00	(0.0, 0.0)
94	SHCG	30.0	Bacteroidia	Nitrosos.	0.00	0.00	0.00	(0.0, 0.0)
95	SHCG	31.0	Alphapro.	Syntrophia	0.00	0.00	0.00	(0.0, 0.0)
96	SHCG	30.0	Alphapro.	Cyano.	0.00	0.00	0.00	(0.0, 0.0)
97	SHCG	30.0	Bacteroidia	Vicinami.	0.00	0.00	0.00	(0.0, 0.0)
98	SHCG	32.0	Gammapro.	Vicinami.	0.00	0.00	0.00	(0.0, 0.0)
99	SHCG	31.0	Alphapro.	Ignavi.	0.00	0.00	0.00	(0.0, 0.0)
100	SHCG	31.0	Alphapro.	Blasto.	0.00	0.00	0.00	(0.0, 0.0)
101	SHCG	31.0	unclass. Proteo.	Vicinami.	0.00	0.00	0.00	(0.0, 0.0)
102	SHCG	31.0	Alphapro.	Oxyphoto.	0.00	0.00	0.00	(0.0, 0.0)
103	SHCG	32.0	Clostridia	Methano.	0.00	0.00	0.00	(0.0, 0.0)
104	SHCG	30.0	Bacteroidia	Ignavi.	0.00	0.00	0.00	(0.0, 0.0)
105	SHCG	30.0	Alphapro.	Oxyphoto.	-0.00	0.00	-0.00	(-0.0, -0.0)
106	SHCG	30.0	Bacteroidia	Nitrospiria	0.00	0.00	0.00	(0.0, 0.0)
107	SHCG	32.0	Gammapro.	MBNT15	0.00	0.00	0.00	(0.0, 0.0)
108	SHCG	30.0	Bacteroidia	Syntrophia	0.00	0.00	0.00	(0.0, 0.0)
109	SHCG	31.0	unclass. Proteo.	Desulfuro.	0.00	0.00	0.00	(0.0, 0.0)
110	SHCG	31.0	Actino.	Corio.	0.00	0.00	0.00	(0.0, 0.0)
111	SHCG	32.0	Gammapro.	Ignavi.	0.00	0.00	0.00	(0.0, 0.0)
112	SHCG	32.0	Gammapro.	Anaerolineae	0.00	0.00	0.00	(0.0, 0.0)
113	SHCG	30.0	Bacteroidia	Desulfuro.	0.00	0.00	0.00	(0.0, 0.0)
114	SHCG	32.0	Gammapro.	Campylo.	0.00	0.00	0.00	(0.0, 0.0)
115	SHCG	31.0	unclass. Proteo.	Fuso.	0.00	0.00	0.00	(0.0, 0.0)
116	SHCG	30.0	Bacteroidia	Fimbrii.	0.00	0.00	0.00	(0.0, 0.0)
117	SHCG	32.0	Gammapro.	Fuso.	0.00	0.00	0.00	(0.0, 0.0)
118	SHCG	30.0	Bacteroidia	Methylomirabilia	0.00	0.00	0.00	(0.0, 0.0)
119	SHCG	30.0	Bacteroidia	Campylo.	0.00	0.00	0.00	(0.0, 0.0)
120	SHCG	30.0	Bacteroidia	Myxococcia	0.00	0.00	0.00	(0.0, 0.0)
121	SHCG	30.0	Alphapro.	Blasto.	0.00	0.00	0.00	(0.0, 0.0)
122	SHCG	32.0	Bacilli	Oxyphoto.	0.00	0.00	0.00	(0.0, 0.0)
123	SHCG	30.0	Bacteroidia	Desulfo.	0.00	0.00	0.00	(0.0, 0.0)
124	SHCG	30.0	Bacteroidia	Polyangia	0.00	0.00	0.00	(0.0, 0.0)
125	SHCG	30.0	Bacteroidia	Fuso.	0.00	0.00	0.00	(0.0, 0.0)
126	SHCG	30.0	Bacteroidia	unclass. Actino.	0.00	0.00	0.00	(0.0, 0.0)
127	SHCG	30.0	Bacteroidia	Methano.	0.00	0.00	0.00	(0.0, 0.0)
128	SHCG	31.0	unclass. Bact.	Corio.	0.00	0.00	0.00	(0.0, 0.0)
129	SHCG	30.0	Bacteroidia	Planctomycetes	0.00	0.00	0.00	(0.0, 0.0)

Continued on next page

TABLE S8: continued

	Model	Time	Source	Target	LOMAR (mean)	var	med	95% CI
130	SHCG	32.0	unclass. Bact.	Verruco.	0.00	0.00	0.00	(0.0, 0.0)
131	SHCG	31.0	Gammapro.	Corio.	0.00	0.00	0.00	(0.0, 0.0)
132	SHCG	30.0	Bacteroidia	Cyano.	0.00	0.00	0.00	(0.0, 0.0)
133	SHCG	32.0	Gammapro.	Oxyphoto.	0.00	0.00	0.00	(0.0, 0.0)
134	SHCG	30.0	Bacteroidia	Blasto.	0.00	0.00	0.00	(0.0, 0.0)

TABLE S9: Relative abundance phenotypic differences (appropriate − suboptimal) of top taxa, before and after 31 weeks PMA.

	UChicago				Boston			
	$<$ 31 weeks		$\geq$ 31 weeks		$<$ 31 weeks		$\geq$ 31 weeks	
Taxa	Diff.	p	Diff.	p	Diff.	p	Diff.	p
Gammaproteo.	0.201	0.013	0.101	0.148	0.345	0.002	0.053	0.374
unclass. Bact.	−0.132	0.036	−0.011	0.734	−0.162	0.055	0.044	0.026
Bacilli	−0.124	0.003	−0.139	0.004	−0.019	0.622	0.002	0.962
Clostridia	0.040	0.266	0.044	0.033	−0.136	0.039	−0.088	0.002
Negativicutes	0.001	0.918	−0.023	0.259	0.031	0.102	0.021	0.305
Bacteroidia	0.050	0.161	0.059	0.012	−0.050	0.382	−0.029	0.186
Actinobacteria	−0.025	0.051	0.010	0.570	−0.007	0.859	−0.000	0.991
unclass. Proteo.	−0.006	0.537	−0.055	0.045	0.000	—	−0.000	0.388
Alphaproteo.	0.001	0.738	0.006	0.409	−0.001	0.344	0.002	0.463
Fusobacteriia	−0.006	0.186	0.009	0.160	−0.000	0.481	−0.006	0.426
Coriobacteriia	0.001	0.241	0.001	0.248	0.001	0.306	0.000	0.297
Vicinamibacteria	−0.000	0.901	−0.000	0.080	−0.000	0.331	0.000	0.551
Verrucomicro.	−0.001	0.225	0.000	0.186	−0.000	0.652	−0.000	0.764
Nitrospiria	−0.000	0.968	−0.000	0.184	−0.000	0.331	0.000	0.930
MBNT15	−0.000	0.631	−0.000	0.166	−0.000	0.331	0.000	0.674
Ignavibacteria	−0.000	0.708	−0.000	0.112	−0.000	0.331	0.000	0.416
Anaerolineae	−0.000	0.779	−0.000	0.139	−0.000	0.423	0.000	0.941
Blastocatellia	0.001	0.335	−0.000	0.170	−0.000	0.331	0.000	0.535
Campylobacteria	−0.000	0.683	0.000	0.403	0.000	—	−0.000	0.685
Desulfuro.	−0.000	0.892	0.000	0.476	0.000	—	0.000	0.688

TABLE S10: Abbreviations of clinical variables associated with MIND cohorts

2_Morbidity	Second morbidity present
abx_consecutive	Longest consecutive days of all antibiotics administered
abx_days	Number of days all antibiotics administered
BHC	Birth head circumference
BPD	Bronchopulmonary dysplasia
BW	Birth weight
Cephalosporins_days	Number of days Cephalosporins administered
days_tpn	Number of days of total parenteral nutrition
DOL_full_feeds	Total enteral feeds, day of life achieved
EGA	Estimated gestational age at birth
Gentamicin_consecutive	Longest consecutive days Gentamicin administered
Gentamicin_days	Number of days Gentamicin administered
HM_percent	Percentage of human milk fed
Len_Stay	Length of NICU stay
Male	Sex
Morbidities	Number of morbidities
Morbidity	Morbidity present
NEC	Necrotizing enterocolitis
Pencillins_days	Number of days Penicillins administered
PMA_dis	Postmenstrual age at NICU discharge
SBI	Severe brain injury
Seizures	Seizures
Sepsis	Sepsis
SR	Severe retinopathy of prematurity
total_enteral	Total amount of enteral feeds
total_formula	Total amount of formula fed
total_HM	Total amount of human milk fed
VD	Vaginal delivery

TABLE S11: Phenotypic groupings of subjects based on SHAP profiles - UChicago cohort

Subject ID	Pheno. grouping	HG
100004	Actinobacteria pref.	appropriate
100051	Actinobacteria pref.	appropriate
100057	Actinobacteria pref.	appropriate
100001	Bacteroidia pref.	appropriate
100003	Bacteroidia pref.	appropriate
100009	Bacteroidia pref.	appropriate
100017	Bacteroidia pref.	appropriate
100018	Bacteroidia pref.	appropriate
100036	Bacteroidia pref.	appropriate
100043	Bacteroidia pref.	appropriate
100063	Bacteroidia pref.	appropriate
100068	Bacteroidia pref.	appropriate
100076	Bacteroidia pref.	appropriate
100002	Null	appropriate
100005	Null	appropriate
100006	Null	appropriate
100019	Null	appropriate
100021	Null	appropriate
100034	Null	appropriate
100050	Null	appropriate
100056	Null	appropriate
100061	Null	appropriate
100064	Null	appropriate
100069	Null	appropriate
100070	Null	appropriate
100078	Null	appropriate
100079	Null	appropriate
100108	Null	appropriate
100032	Actinobacteria pref.	suboptimal
100014	Bacteroidia pref.	suboptimal
100020	Bacteroidia pref.	suboptimal
100025	Bacteroidia pref.	suboptimal
100052	Bacteroidia pref.	suboptimal
100060	Bacteroidia pref.	suboptimal
100066	Bacteroidia pref.	suboptimal
100083	Bacteroidia pref.	suboptimal
100090	Bacteroidia pref.	suboptimal
100007	Null	suboptimal
100010	Null	suboptimal
100011	Null	suboptimal
100012	Null	suboptimal
100015	Null	suboptimal
100016	Null	suboptimal
100026	Null	suboptimal
100028	Null	suboptimal
100033	Null	suboptimal
100041	Null	suboptimal
100046	Null	suboptimal
100049	Null	suboptimal
100053	Null	suboptimal
100054	Null	suboptimal
100055	Null	suboptimal
100058	Null	suboptimal
100062	Null	suboptimal
100065	Null	suboptimal
100075	Null	suboptimal

Continued on next page

TABLE S11: continued

Subject ID	Pheno. grouping	HG
100084	Null	suboptimal
100091	Null	suboptimal

TABLE S12: Phenotypic groupings of subjects based on SHAP profiles - Boston cohort

Subject ID	Pheno. grouping	HG
136000	Bacteroidia pref.	appropriate
136001	Bacteroidia pref.	appropriate
136005	Bacteroidia pref.	appropriate
136007	Bacteroidia pref.	appropriate
136010	Bacteroidia pref.	appropriate
136012	Bacteroidia pref.	appropriate
136026	Bacteroidia pref.	appropriate
136033	Bacteroidia pref.	appropriate
136039	Bacteroidia pref.	appropriate
136041	Bacteroidia pref.	appropriate
136042	Bacteroidia pref.	appropriate
136043	Bacteroidia pref.	appropriate
136045	Bacteroidia pref.	appropriate
136052	Bacteroidia pref.	appropriate
136021	Null	appropriate
136038	Null	appropriate
136040	Null	appropriate
136044	Null	appropriate
136006	Bacteroidia pref.	suboptimal
136015	Bacteroidia pref.	suboptimal
136025	Bacteroidia pref.	suboptimal
136030	Bacteroidia pref.	suboptimal
136031	Bacteroidia pref.	suboptimal
136032	Bacteroidia pref.	suboptimal
136035	Bacteroidia pref.	suboptimal
136037	Bacteroidia pref.	suboptimal
136046	Bacteroidia pref.	suboptimal
136050	Bacteroidia pref.	suboptimal
136047	Null	suboptimal
136051	Null	suboptimal

TABLE S13: Performance measures for classification at 32 weeks PMA

UChicago						
fpr	tpr	ppv	acc	npv	LR+	LR-
0.02	0.379 ± 0.125	0.953 ± 0.024	0.669 ± 0.065	0.598 ± 0.038	18.957 ± 6.274	0.634 ± 0.128
0.04	0.469 ± 0.143	0.928 ± 0.024	0.706 ± 0.074	0.629 ± 0.049	11.719 ± 3.581	0.553 ± 0.149
0.06	0.525 ± 0.111	0.906 ± 0.02	0.726 ± 0.058	0.65 ± 0.041	8.758 ± 1.856	0.505 ± 0.118
0.08	0.581 ± 0.094	0.889 ± 0.018	0.745 ± 0.048	0.673 ± 0.037	7.264 ± 1.171	0.455 ± 0.102
0.1	0.617 ± 0.074	0.871 ± 0.014	0.754 ± 0.038	0.688 ± 0.031	6.174 ± 0.742	0.425 ± 0.082
0.12	0.652 ± 0.054	0.855 ± 0.012	0.762 ± 0.028	0.703 ± 0.024	5.431 ± 0.446	0.396 ± 0.061
0.14	0.68 ± 0.049	0.841 ± 0.011	0.767 ± 0.025	0.715 ± 0.023	4.857 ± 0.348	0.372 ± 0.057
0.16	0.709 ± 0.049	0.828 ± 0.01	0.772 ± 0.025	0.73 ± 0.024	4.431 ± 0.307	0.346 ± 0.059
0.18	0.736 ± 0.051	0.817 ± 0.01	0.777 ± 0.027	0.744 ± 0.026	4.09 ± 0.286	0.322 ± 0.063
0.2	0.759 ± 0.052	0.806 ± 0.01	0.779 ± 0.027	0.756 ± 0.028	3.794 ± 0.261	0.302 ± 0.065
Boston						
fpr	tpr	ppv	acc	npv	LR+	LR-
0.02	0.248 ± 0.158	0.934 ± 0.023	0.601 ± 0.082	0.551 ± 0.053	12.393 ± 7.899	0.767 ± 0.161
0.04	0.298 ± 0.131	0.901 ± 0.022	0.618 ± 0.068	0.563 ± 0.046	7.453 ± 3.278	0.731 ± 0.137
0.06	0.344 ± 0.118	0.874 ± 0.021	0.632 ± 0.061	0.574 ± 0.044	5.733 ± 1.975	0.698 ± 0.126
0.08	0.374 ± 0.109	0.85 ± 0.019	0.638 ± 0.056	0.58 ± 0.043	4.675 ± 1.361	0.68 ± 0.118
0.1	0.403 ± 0.099	0.832 ± 0.019	0.643 ± 0.051	0.586 ± 0.041	4.032 ± 0.992	0.663 ± 0.11
0.12	0.437 ± 0.103	0.815 ± 0.018	0.651 ± 0.054	0.595 ± 0.045	3.645 ± 0.862	0.639 ± 0.118
0.14	0.463 ± 0.095	0.799 ± 0.017	0.654 ± 0.049	0.6 ± 0.042	3.304 ± 0.679	0.625 ± 0.111
0.16	0.485 ± 0.087	0.784 ± 0.016	0.656 ± 0.045	0.605 ± 0.041	3.033 ± 0.543	0.613 ± 0.103
0.18	0.511 ± 0.081	0.772 ± 0.015	0.66 ± 0.042	0.611 ± 0.04	2.837 ± 0.449	0.597 ± 0.099
0.2	0.533 ± 0.079	0.761 ± 0.015	0.662 ± 0.041	0.617 ± 0.04	2.666 ± 0.395	0.584 ± 0.099

TABLE S14: Q-net model instances generating the stated results*

Q-net model example	Link
Appropriate cohort model, basic example	https://zenodo.org/record/7453697/files/appr.joblib
Suboptimal cohort model, basic example	https://zenodo.org/record/7453697/files/sub.joblib
Appropriate cohort model, classification example	https://zenodo.org/record/7453697/files/classification_ahctg.joblib
Combined (both cohort) model, classification example	https://zenodo.org/record/7453697/files/classification_combined.joblib
Suboptimal cohort model, classification example	https://zenodo.org/record/7453697/files/classification_shctg.joblib
Appropriate cohort model, feeding example	https://zenodo.org/record/7453697/files/feeding_ahctg.joblib
Combined (both cohort) model, feeding example	https://zenodo.org/record/7453697/files/feeding_combined.joblib
Suboptimal cohort model, feeding example	https://zenodo.org/record/7453697/files/feeding_shctg.joblib
Appropriate cohort model, hypothesis example	https://zenodo.org/record/7453697/files/hypotheses_ahctg.joblib
Combined (both cohort) model, hypothesis example	https://zenodo.org/record/7453697/files/hypotheses_combined.joblib
Suboptimal cohort model, hypothesis example	https://zenodo.org/record/7453697/files/hypotheses_shctg.joblib

Appropriate cohort model, SHAP examples	https://zenodo.org/record/7942501/files/shap-ahcg-1.joblib<br>https://zenodo.org/record/7942501/files/shap-ahcg-2.joblib<br>https://zenodo.org/record/7942501/files/shap-ahcg-3.joblib<br>https://zenodo.org/record/7942501/files/shap-ahcg-4.joblib<br>https://zenodo.org/record/7942501/files/shap-ahcg-5.joblib<br>https://zenodo.org/record/7942501/files/shap-ahcg-6.joblib<br>https://zenodo.org/record/7942501/files/shap-ahcg-7.joblib<br>https://zenodo.org/record/7942501/files/shap-ahcg-8.joblib<br>https://zenodo.org/record/7942501/files/shap-ahcg-9.joblib<br>https://zenodo.org/record/7942501/files/shap-ahcg-10.joblib
Subpotimal cohort model, SHAP examples	https://zenodo.org/record/7942501/files/shap-shcg-1.joblib<br>https://zenodo.org/record/7942501/files/shap-shcg-2.joblib<br>https://zenodo.org/record/7942501/files/shap-shcg-3.joblib<br>https://zenodo.org/record/7942501/files/shap-shcg-4.joblib<br>https://zenodo.org/record/7942501/files/shap-shcg-5.joblib<br>https://zenodo.org/record/7942501/files/shap-shcg-6.joblib<br>https://zenodo.org/record/7942501/files/shap-shcg-7.joblib<br>https://zenodo.org/record/7942501/files/shap-shcg-8.joblib<br>https://zenodo.org/record/7942501/files/shap-shcg-9.joblib<br>https://zenodo.org/record/7942501/files/shap-shcg-10.joblib

⋆ Regeneration of the Q-nets can produce slightly different models with respect to connectivities and generation probabilities since the inference algorithm is not deterministic. The performance results should still be within confidence limits.

TABLE S15: Documented dependencies between bacterial classes in the human gut microbiome (⋆Negative association; †Positive association)

Year	Associating Bacterial Classes	First Author et al.[]
2012	Actinobacteria– Bacteroidia	Turroni et al.[100]†
2013	Bacteroidia– Clostridia (mouse)	Sybille et al.[101]⋆
2020	Bacteroidia– Verrucomicrobia (mouse)	Xu et al.[102]†
2016	Actinobacteria– Clostridia	Rivière et al.[103]†
2016	Bacteroidia– Gammaproteobacteria	Serrazanetti et al.[104] ⋆
2016	Bacteroidia– Bacilli	Serrazanetti et al.[104] ⋆
2015	Bacteroidia– Clostridia	Zhang et al.[105] ⋆
2019	Bacteroidia– Verrucomicrobia	Zhang et al.[106] †

TABLE S16: Total number of model re-generations used.

Forecasting	Appropriate: 26 model instances	11 runs (in sample)<br>15 runs (out of sample)
	Suboptimal: 26 model instances	11 runs (in sample)<br>15 runs (out of sample)
Classification/risk computation	Appropriate: 240 model instances<br>Suboptimal: 240 model instances	20 runs$\times$12 weeks<br>20 runs$\times$12 weeks
LOMAR	Appropriate: 10 model instances<br>Suboptimal: 10 model instances	
SHAP/Clinical SHAP analysis	Appropriate: 10 model instances<br>Suboptimal: 10 model instances	100 runs (clinical SHAP)

REFERENCES AND NOTES

1. J. F. Cryan, T. G. Dinan, Mind-altering microorganisms: The impact of the gut microbiota on brain and behaviour. *Nat. Rev. Neurosci.* **13**, 701–712 (2012).

2. M. E. DiBartolomeo, E. C. Claud, The developing microbiome of the preterm infant. *Clin. Ther.* **38**, 733–739 (2016).

3. L. Lu, Y. Yu, Y. Guo, Y. Wang, E. B. Chang, E. C. Claud, Transcriptional modulation of intestinal innate defense/inflammation genes by preterm infant microbiota in a humanized gnotobiotic mouse model. *PLOS ONE* **10**, e0124504 (2015).

4. J. R. Baranowski, E. C. Claud, Necrotizing enterocolitis and the preterm infant microbiome. *Adv. Exp. Med. Biol.* **1125**, 25–36 (2019).

5. A. L. Carlson, K. Xia, M. A. Azcarate-Peril, B. D. Goldman, M. Ahn, M. A. Styner, A. L. Thompson, X. Geng, J. H. Gilmore, R. C. Knickmeyer, Infant gut microbiome associated with cognitive development. *Biol. Psychiatry* **83**, 148–159 (2018).

6. A. Burokas, R. D. Moloney, T. G. Dinan, J. F. Cryan, in *Advances in Applied Microbiology* (Elsevier, 2015), vol. 91, pp. 1–62.

7. J. Lu, S. Synowiec, L. Lu, Y. Yu, T. Bretherick, S. Takada, V. Yarnykh, J. Caplan, M. Caplan, E. C. Claud, A. Drobyshevsky, Microbiota influence the development of the brain and behaviors in C57BL/6J mice. *PLOS ONE* **13**, e0201829 (2018).

8. J. Lu, E. C. Claud, Connection between gut microbiome and brain development in preterm infants. *Dev. Psychobiol.* **61**, 739–751 (2019).

9. D.-W. Kang, J. G. Park, Z. E. Ilhan, G. Wallstrom, J. LaBaer, J. B. Adams, R. Krajmalnik-Brown, Reduced incidence of prevotella and other fermenters in intestinal microflora of autistic children. *PLOS ONE* **8**, e68322 (2013).

10. E. Y. Hsiao, S. W. McBride, S. Hsien, G. Sharon, E. R. Hyde, T. McCue, J. A. Codelli, J. Chow, S. E. Reisman, J. F. Petrosino, P. H. Patterson, S. K. Mazmanian, Microbiota modulate

behavioral and physiological abnormalities associated with neurodevelopmental disorders. *Cell* **155**, 1451–1463 (2013).

11. K. Oliphant, M. Ali, M. D'Souza, P. D. Hughes, D. Sulakhe, A. Z. Wang, B. Xie, R. Yeasin, M. E. Msall, B. Andrews, E. C. Claud, Bacteroidota and lachnospiraceae integration into the gut microbiome at key time points in early life are linked to infant neurodevelopment. *Gut Microbes* **13**, 1997560 (2021).

12. T. Hooven, Y. C. Lin, A. Salleb-Aouissi, Multiple instance learning for predicting necrotizing enterocolitis in premature infants using microbiome data. *Proc. ACM Conf. Health Inference Learn.* **2020**, 99–109 (2020).

13. M. R. Olm, N. Bhattacharya, A. Crits-Christoph, B. A. Firek, R. Baker, Y. S. Song, M. J. Morowitz, J. F. Banfield, Necrotizing enterocolitis is preceded by increased gut bacterial replication, *Klebsiella*, and fimbriae-encoding bacteria. *Sci. Adv.* **5**, eaax5727 (2019).

14. H. E. Laue, S. A. Korrick, E. R. Baker, M. R. Karagas, J. C. Madan, Prospective associations of the infant gut microbiome and microbial function with social behaviors related to autism at age 3 years. *Sci. Rep.* **10**, 15515 (2020).

15. M. S. Matchado, M. Lauber, S. Reitmeier, T. Kacprowski, J. Baumbach, D. Haller, M. List, Network analysis methods for studying microbial communities: A mini review. *Comput. Struct. Biotechnol. J.* **19**, 2687–2698 (2021).

16. M. A. Fischbach, Microbiome: Focus on causation and mechanism. *Cell* **174**, 785–790 (2018).

17. K. Faust, J. Raes, Conet app: Inference of biological association networks using cytoscape. *F1000Res.* **5**, 1519 (2016).

18. Y. Deng, Y.H. Jiang, Y. Yang, Z. He, F. Luo, J. Zhou, Molecular ecological network analyses. *BMC Bioinformatics* **13**, 113 (2012).

19. J. Friedman, E. J. Alm, Inferring correlation networks from genomic survey data. *PLoS Comput. Biol.* **8**, e1002687 (2012).

20. H. Fang, C. Huang, H. Zhao, M. Deng, Cclasso: Correlation inference for compositional data through lasso. *Bioinformatics* **31**, 3172–3180 (2015).

21. Y. Ban, L. An, H. Jiang, Investigating microbial co-occurrence patterns based on metagenomic compositional data. *Bioinformatics* **31**, 3322–3329 (2015).

22. P. Gajer, R. M. Brotman, G. Bai, J. Sakamoto, U. M. E. Schütte, X. Zhong, S. S. K. Koenig, L. Fu, Z. (S.) Ma, X. Zhou, Z. Abdo, L. J. Forney, J. Ravel, Temporal dynamics of the human vaginal microbiota. *Sci. Transl. Med.* **4**, 132ra52 (2012).

23. P. S. La Rosa, B. B. Warner, Y. Zhou, G. M. Weinstock, E. Sodergren, C. M. Hall-Moore, H. J. Stevens, W. E. Bennett Jr, N. Shaikh, L. A. Linneman, J. A. Hoffmann, A. Hamvas, E. Deych, B. A. Shands, W. D. Shannon, P. I. Tarr, Patterned progression of bacterial populations in the premature infant gut. *Proc. Natl. Acad. Sci.* **111**, 12522–12527 (2014).

24. T. Gibson, G. Gerber, Robust and scalable models of microbiome dynamics. *PMLR* **80**, 1763–1772 (2018).

25. M. J. McGeachie, J. E. Sordillo, T. Gibson, G. M. Weinstock, Y.Y. Liu, D. R. Gold, S. T. Weiss, A. Litonjua, Longitudinal prediction of the infant gut microbiome with dynamic bayesian networks. *Sci. Rep.* **6**, 20359 (2016).

26. J. Lugo-Martinez, D. Ruiz-Perez, G. Narasimhan, Z. Bar-Joseph, Dynamic interaction network inference from longitudinal microbiome data. *Microbiome* **7**, 54 (2019).

27. T. A. Joseph, A. P. Pasarkar, I. Pe'er, Efficient and accurate inference of mixed microbial population trajectories from longitudinal count data. *Cell Syst.* **10**, 463–469.e6 (2020).

28. D. Ruiz-Perez, J. Lugo-Martinez, N. Bourguignon, K. Mathee, B. Lerner, Z. Bar-Joseph, G. Narasimhan, Dynamic bayesian networks for integrating multi-omics time series microbiome data. *mSystems* **6**, e01105-20(2021).

29. V. Bucci, B. Tzen, N. Li, M. Simmons, T. Tanoue, E. Bogart, L. Deng, V. Yeliseyev, M. L. Delaney, Q. Liu, B. Olle, R. R. Stein, K. Honda, L. Bry, G. K. Gerber, Mdsine: Microbial dynamical systems inference engine for microbiome time-series analyses. *Genome Biol.* **17**, 121 (2016).

30. M. Alshawaqfeh, E. Serpedin, A. B. Younes, Inferring microbial interaction networks from metagenomic data using sglv-ekf algorithm. *BMC Genomics* **18**, 228 (2017).

31. X. Gao, B.-T. Huynh, D. Guillemot, P. Glaser, L. Opatowski, Inference of significant microbial interactions from longitudinal metagenomics data. *Front. Microbiol.* **9**, 2319 (2018).

32. K. Raghuram, J. Yang, P. T. Church, Z. Cieslak, A. Synnes, A. Mukerji, P. S. Shah, for the Canadian Neonatal Network, Canadian Neonatal Follow-Up Network Investigators, Head growth trajectory and neurodevelopmental outcomes in preterm neonates. *Pediatrics* **140**, e20170216 (2017).

33. K. C. K. Kuban, E. N. Allred, T Michael O'Shea, N. Paneth, S. Westra, C. Miller, N. P. Rosman, A. Leviton, Developmental correlates of head circumference at birth and two years in a cohort of extremely low gestational age newborns. *J. Pediatr.* **155**, 344–349.e3 (2009).

34. V. Neubauer, T. Fuchs, E. Griesmaier, K. Kager, U. Pupp-Peglow, U. Kiechl-Kohlendorfer, Poor postdischarge head growth is related to a 10% lower intelligence quotient in very preterm infants at the chronological age of five years. *Acta Paediatr.* **105**, 501–507 (2016).

35. E. G. Cordova, S. Cherkerzian, K. Bell, K. E. Joung, C. T. Collins, M. Makrides, J. Gould, P. J. Anderson, M. B. Belfort, Association of poor postnatal growth with neurodevelopmental impairment in infancy and childhood: Comparing the fetus and the healthy preterm infant references. *J. Pediatr.* **225**, 37–43.e5 (2020).

36. M. Hack, N. Breslau, B. Weissman, D. Aram, N. Klein, E. Borawski, Effect of very low birth weight and subnormal head size on cognitive abilities at school age. *N. Eng. J. Med.* **325**, 231–237 (1991).

37. M. B. Belfort, S. L. Rifas-Shiman, T. Sullivan, C. T. Collins, A. J. McPhee, P. Ryan, K. P. Kleinman, M. W. Gillman, R. A. Gibson, M. Makrides, Infant growth before and after term: Effects on neurodevelopment in preterm infants. *Pediatrics* **128**, e899–e906 (2011).

38. W. Walters, E. R. Hyde, D. Berg-Lyons, G. Ackermann, G. Humphrey, A. Parada, J. A. Gilbert, J. K. Jansson, J. G. Caporaso, J. A. Fuhrman, A. Apprill, R. Knight, Improved bacterial 16s rrna gene (v4 and v4-5) and fungal internal transcribed spacer marker gene primers for microbial community surveys. *mSystems* **1**, e00009–15 (2016).

39. J. G. Caporaso, C. L. Lauber, W. A. Walters, D. Berg-Lyons, J. Huntley, N. Fierer, S. M. Owens, J. Betley, L. Fraser, M. Bauer, N. Gormley, J. A. Gilbert, G. Smith, R. Knight, Ultra-high-throughput microbial community analysis on the illumina hiseq and miseq platforms. *ISME J.* **6**, 1621–1624 (2012).

40. T. R. Fenton, J. H. Kim, A systematic review and meta-analysis to revise the fenton growth chart for preterm infants. *BMC Pediatr.* **13**, 59 (2013).

41. T. Hothorn, K. Hornik, A. Zeileis, Unbiased recursive partitioning: A conditional inference framework. *J. Comput. Graph. Stat.* **15**, 651–674 (2006).

42. A. Sarda-Espinosa, S. Subbiah, T. Bartz-Beielstein, Conditional inference trees for knowledge extraction from motor health condition data. *Eng. Appl. Artif. Intel.* **62**, 26–37 (2017).

43. S. Geman, D. Geman, Stochastic relaxation, gibbs distributions, and the bayesian restoration of images. *IEEE Trans. Pattern Anal. Mach. Intell.* **PAMI-6**, 721–741 (1984).

44. G. Casella, E. I. George, Explaining the gibbs sampler. *Am. Stat.* **46**, 167–174 (1992).

45. P. M. Dietz, L. J. England, W. M. Callaghan, M. Pearl, M. L. Wier, M. Kharrazi, A comparison of lmp-based and ultrasound-based estimates of gestational age using linked california livebirth and prenatal screening records. *Paediatr. Perinat. Epidemiol.* **21**, 62–71 (2007).

46. J. Lopez, A. Grinspan, Fecal microbiota transplantation for inflammatory bowel disease. *Gastroenterol. Hepatol.* **12**, 374–379 (2016).

47. T. M. Cover, J. A. Thomas, *Elements of Information Theory (Wiley Series in Telecommunications and Signal Processing)* (Wiley-Interscience, 2006).

48. S. M. Lundberg, S.-I. Lee, in *Advances in Neural Information Processing Systems* 30, I. Guyon, et al., Eds. (Curran Associates, Inc., 2017), pp. 4765–4774.

49. S. M. Lundberg, B. Nair, M. S. Vavilala, M. Horibe, M. J. Eisses, T. Adams, D. E. Liston, D. K.W. Low, S.F. Newman, J. Kim, S.I. Lee, Explainable machine-learning predictions for the prevention of hypoxaemia during surgery. *Nat. Biomed. Eng.* **2**, 749–760 (2018).

50. E. Štrumbelj, I. Kononenko, Explaining prediction models and individual predictions with feature contributions. *Knowl. Inf. Syst.* **41**, 647–665 (2014).

51. A. E. Roth, *The Shapley Value: Essays in Honor of Lloyd S. Shapley* (Cambridge University Press, 2009).

52. E. Zaura, B. J. Keijser, S. M. Huse, W. Crielaard, Defining the healthy "core microbiome" of oral microbial communities. *BMC Microbiol.* **9**, 259 (2009).

53. C. A. Lozupone, J. I. Stombaugh, J. I. Gordon, J. K. Jansson, R. Knight, Diversity, stability and resilience of the human gut microbiota. *Nature* **489**, 220–230 (2012).

54. T. Yatsunenko, F. E. Rey, M. J. Manary, I. Trehan, M. G. Dominguez-Bello, M. Contreras, M. Magris, G. Hidalgo, R. N. Baldassano, A. P. Anokhin, A. C. Heath, B. Warner, J. Reeder, J. Kuczynski, J. G. Caporaso, C. A. Lozupone, C. Lauber, J. C. Clemente, D. Knights, R. Knight, J. I. Gordon, Human gut microbiome viewed across age and geography. *Nature* **486**, 222–227 (2012).

55. A. Prehn-Kristensen, A. Zimmermann, L. Tittmann, W. Lieb, S. Schreiber, L. Baving, A. Fischer, Reduced microbiome alpha diversity in young patients with adhd. *PLOS ONE* **13**, e0200728 (2018).

56. M. Fox, S. M. Lee, K. S. Wiley, V. Lagishetty, C. A. Sandman, J. P. Jacobs, L. M. Glynn, Development of the infant gut microbiome predicts temperament across the first year of life. *Dev. Psychopathol.* **34**, 1914–1925 (2022).

57. Y. Wang, X. Chen, Y. Yu, Y. Liu, Q. Zhang, J. Bai, Association between gut microbiota and infant's temperament in the first year of life in a Chinese birth cohort. *Microorganisms* **8**, 753 (2020).

58. I. Acuña, T. Cerdó, A. Ruiz, F. J. Torres-Espínola, A. López-Moreno, M. Aguilera, A. Suárez, C. Campoy, Infant gut microbiota associated with fine motor skills. *Nutrients* **13**, 1673 (2021).

59. S. Abuaish, N. M. al-Otaibi, T. S. Abujamel, S. A. Alzahrani, S. M. Alotaibi, Y. A. AlShawakir, K. Aabed, A. el-Ansary, Fecal transplant and *Bifidobacterium* treatments modulate gut *Clostridium* bacteria and rescue social impairment and hippocampal BDNF expression in a rodent model of autism. *Brain Sci.* **11**, 1038 (2021).

60. B. Ma, S. Sundararajan, G. Nadimpalli, M. France, E. McComb, L. Rutt, J. M. Lemme-Dumit, E. Janofsky, L. S. Roskes, P. Gajer, L. Fu, H. Yang, M. Humphrys, L. J. Tallon, L. Sadzewicz, M. F. Pasetti, J. Ravel, R. M. Viscardi, Highly specialized carbohydrate metabolism capability in *Bifidobacterium* strains associated with intestinal barrier maturation in early preterm infants. *MBio* **13**, e0129922 (2022).

61. J. Samara, S. Moossavi, B. Alshaikh, V. A. Ortega, V. K. Pettersen, T. Ferdous, S. L. Hoops, A. Soraisham, J. Vayalumkal, D. Dersch-Mills, J. S. Gerber, S. Mukhopadhyay, K. Puopolo, T. A. Tompkins, D. Knights, J. Walter, H. Amin, M.C. Arrieta, Supplementation with a probiotic mixture accelerates gut microbiome maturation and reduces intestinal inflammation in extremely preterm infants. *Cell Host Microbe* **30**, 696–711.e5 (2022).

62. R. L. Morgan, G. A. Preidis, P. C. Kashyap, A. V. Weizman, B. Sadeghirad, Y. Chang, I. D. Florez, F. Foroutan, S. Shahid, D. Zeraatkar, Probiotics reduce mortality and morbidity in preterm, low-birth-weight infants: A systematic review and network meta-analysis of randomized trials. *Gastroenterology* **159**, 467–480 (2020).

63. A. Acuna-Gonzalez, M. Kujawska, M. Youssif, T. Atkinson, S. Grundy, A. Hutchison, C. Tremlett, P. Clarke, L. J. Hall, Bifidobacterium bacteraemia is rare with routine probiotics use in preterm infants: A further case report with literature review. *Anaerobe* **80**, 102713 (2023).

64. S. E. Jacobs, L. Hickey, S. Donath, G. F. Opie, P. J. Anderson, S. M. Garland, J. L. Y. Cheong, ProPremsStudy Groups, Probiotics, prematurity and neurodevelopment: Follow-up of a randomised trial. *BMJ Paediatrics Open* **1**, e000176 (2017).

65. S. Khanna, C. S. Kraft, Fecal microbiota transplantation: Tales of caution. *Clin. Infect. Dis.* **72**, e881–e882 (2020).

66. L. Michailidis, A. C. Currier, M. Le, D. R. Flomenhoft, Adverse events of fecal microbiota transplantation: A meta-analysis of high-quality studies. *Ann. Gastroenterol.* **34**, 802 (2021).

67. A. Ohishi, S. Takahashi, Y. Ito, Y. Ohishi, K. Tsukamoto, Y. Nanba, N. Ito, S. Kakiuchi, A. Saitoh, M. Morotomi, T. Nakamura, Bifidobacterium septicemia associated with postoperative probiotic therapy in a neonate with omphalocele. *J. Pediatr.* **156**, 679–681 (2010).

68. E. Weber, Q. Reynaud, F. Suy, A. Gagneux-Brunon, A. Carricajo, A. Guillot, E. Botelho-Nevers, Bifidobacterium species bacteremia: Risk factors in adults and infants. *Clin. Infect. Dis.* **61**, 482–484 (2015).

69. W. Xu, M. P. Judge, K. Maas, N. Hussain, J. M. McGrath, W. A. Henderson, X. Cong, Systematic review of the effect of enteral feeding on gut microbiota in preterm infants *J. Obstet. Gynecol. Neonatal. Nurs.* **47** 451–463 (2018).

70. M. Reyman, M. A. van Houten, R. L. Watson, M. L. J. N. Chu, K. Arp, W. J. de Waal, I. Schiering, F. B. Plötz, R. J. L. Willems, W. van Schaik, E. A. M. Sanders, D. Bogaert, Effects of early-life antibiotics on the developing infant gut microbiome and resistome: A randomized trial. *Nat. Commun.* **13**, 893 (2022).

71. M. K. Gibson, T. S. Crofts, G. Dantas, Antibiotics and the developing infant gut microbiota and resistome. *Curr. Opin. Microbiol.* **27**, 51–56 (2015).

72. G. D. P. Coelho, L. F. A. Ayres, D. S. Barreto, B. D. Henriques, M. R. M. C. Prado, C. M. D. Passos, Acquisition of microbiota according to the type of birth: An integrative review. *Rev. Lat. Am. Enfermagem* **29**, e3446 (2021).

73. E. Jašarević, K. E. Morrison, T. L. Bale, Sex differences in the gut microbiome–brain axis across the lifespan. *Philos. Trans. R. Soc. Lond. B Biol. Sci.* **371**, 20150122 (2016).

74. X. Hu, Y. Chang, Z. Wang, W. Bao, Z. Li, Altered gut microbiota is associated with feeding intolerance in preterm infants. *Turk. J. Pediatr.* **63**, 206–217 (2021).

75. L. Liu, D. Ao, X. Cai, P. Huang, N. Cai, S. Lin, B. Wu, Early gut microbiota in very low and extremely low birth weight preterm infants with feeding intolerance: A prospective case-control study. *J. Microbiol.* **60**, 1021–1031 (2022).

76. A. Mitha, S. S. Kruth, S. Bjurman, A. Rakow, S. Johansson, Neonatal morbidities and feeding tolerance outcomes in very preterm infants, before and after introduction of probiotic supplementation. *Nutrients* **14**, 3646 (2022).

77. B. Alshaikh, J. Samara, S. Moossavi, T. Ferdous, A. Soraisham, D. Dersch-Mills, M.-C. Arrieta, H. Amin, Multi-strain probiotics for extremely preterm infants: A randomized controlled trial. *Pediatr. Res.* **92**, 1663–1670 (2022).

78. L.-X. Qiao, W.-Y. Zhu, H.-Y. Zhang, H. Wang, Effect of early administration of probiotics on gut microflora and feeding in pre-term infants: A randomized controlled trial. *J. Matern. Fetal Neonatal Med.* **30**, 13–16 (2017).

79. T. Zhao, H.-M. Feng, B. Caicike, Y.-P. Zhu, Investigation into the current situation and analysis of the factors influencing extrauterine growth retardation in preterm infants. *Front. Pediatr.* **9**, 643387 (2021).

80. S. P. Bae, E.K. Kim, J. Yun, Y. M. Yoon, S. H. Shin, S. Y. Park, Retinopathy of prematurity requiring treatment is closely related to head growth during neonatal intensive care unit hospitalization in very low birth weight infants. *Neonatology* **119**, 176–183 (2022).

81. B. J. Callahan, P. J. McMurdie, M. J. Rosen, A. W. Han, A. J. A. Johnson, S. P. Holmes, Dada2: High-resolution sample inference from illumina amplicon data. *Nat. Methods* **13**, 581–583 (2016).

82. E. Bolyen, J. R. Rideout, M. R. Dillon, N. A. Bokulich, C. C. Abnet, G. A. al-Ghalith, H. Alexander, E. J. Alm, M. Arumugam, F. Asnicar, Y. Bai, J. E. Bisanz, K. Bittinger, A. Brejnrod, C. J. Brislawn, C. T. Brown, B. J. Callahan, A. M. Caraballo-Rodríguez, J. Chase, E. K. Cope, R. da Silva, C. Diener, P. C. Dorrestein, G. M. Douglas, D. M. Durall, C. Duvallet, C. F. Edwardson, M. Ernst, M. Estaki, J. Fouquier, J. M. Gauglitz, S. M. Gibbons, D. L. Gibson, A. Gonzalez, K. Gorlick, J. Guo, B. Hillmann, S. Holmes, H. Holste, C. Huttenhower, G. A. Huttley, S. Janssen, A. K. Jarmusch, L. Jiang, B. D. Kaehler, K. B. Kang, C. R. Keefe, P. Keim, S. T. Kelley, D. Knights, I. Koester, T. Kosciolek, J. Kreps, M. G. I. Langille, J. Lee, R. Ley, Y.X. Liu, E. Loftfield, C. Lozupone, M. Maher, C. Marotz, B. D. Martin, D. McDonald, L. J. McIver, A. V. Melnik, J. L. Metcalf, S. C. Morgan, J. T. Morton, A. T. Naimey, J. A. Navas-Molina, L. F. Nothias, S. B. Orchanian, T. Pearson, S. L. Peoples, D. Petras, M. L. Preuss, E. Pruesse, L. B. Rasmussen, A. Rivers, M. S. Robeson II, P. Rosenthal, N. Segata, M. Shaffer, A. Shiffer, R. Sinha, S. J. Song, J. R. Spear, A. D. Swafford, L. R. Thompson, P. J. Torres, P. Trinh, A. Tripathi, P. J. Turnbaugh, S. Ul-Hasan, J. J. J. van der Hooft, F. Vargas, Y. Vázquez-Baeza, E. Vogtmann, M. von Hippel, W. Walters, Y. Wan, M. Wang, J. Warren, K. C. Weber, C. H. D. Williamson, A. D. Willis, Z. Z. Xu, J. R. Zaneveld, Y. Zhang, Q. Zhu, R. Knight, J. G. Caporaso, Reproducible, interactive, scalable and extensible microbiome data science using qiime 2. *Nat. Biotechnol.* **37**, 852–857 (2019).

83. A. Murali, A. Bhargava, E. S. Wright, Idtaxa: A novel approach for accurate taxonomic classification of microbiome sequences. *Microbiome* **6**, 140 (2018).

84. D. H. Parks, M. Chuvochina, D. W. Waite, C. Rinke, A. Skarshewski, P.A. Chaumeil, P. Hugenholtz, A standardized bacterial taxonomy based on genome phylogeny substantially revises the tree of life. *Nat. Biotechnol.* **36**, 996–1004 (2018).

85. S. F. Altschul, W. Gish, W. Miller, E. W. Myers, D. J. Lipman, Basic local alignment search tool. *J. Mol. Biol.* **215**, 403–410 (1990).

86. T. M. Cover, J. A. Thomas, *Elements of Information Theory* (Wiley-Interscience, 1991).

87. B. Fuglede, F. Topsoe, in *Proceedings of the International Symposium on Information Theory, 2004* (IEEE, 2004), p. 31.

88. J. Besag, Spatial interaction and the statistical analysis of lattice systems. *J. R. Stat. Soc. B. Methodol.* **36**, 192–225 (1974).

89. A. E. Gelfand, A. F. Smith, Sampling-based approaches to calculating marginal densities. *J. Am. Stat. Assoc.* **85**, 398–409 (1990).

90. R Core Team, *R: A Language and Environment for Statistical Computing* (R Foundation for Statistical Computing, 2021).

91. D. Quesada, dbnR: Dynamic Bayesian Network Learning and Inference (2022). R package version 0.7.8.

92. M. Scutari, Learning Bayesian networks with the bnlearn R package. arXiv:0908.3817 [stat.ML] (2009).

93. N. M. Vogt, R. L. Kerby, Kimberly A Dill-Mc Farland, S. J. Harding, A. P. Merluzzi, S. C. Johnson, C. M. Carlsson, S. Asthana, H. Zetterberg, K. Blennow, B. B. Bendlin, F. E. Rey, Gut microbiome alterations in Alzheimer's disease. *Sci. Rep.* **7**, 13537 (2017).

94. S. Jangi, R. Gandhi, L. M. Cox, N. Li, F. von Glehn, R. Yan, B. Patel, M. A. Mazzola, S. Liu, B. L. Glanz, S. Cook, S. Tankou, F. Stuart, K. Melo, P. Nejad, K. Smith, B. D. Topçuolu, J. Holden, P. Kivisäkk, T. Chitnis, P. L. De Jager, F. J. Quintana, G. K. Gerber, L. Bry, H. L. Weiner, Alterations of the human gut microbiome in multiple sclerosis. *Nat. Commun.* **7**, 12015 (2016).

95. H. Jiang, Z. Ling, Y. Zhang, H. Mao, Z. Ma, Y. Yin, W. Wang, W. Tang, Z. Tan, J. Shi, L. Li, B. Ruan, Altered fecal microbiota composition in patients with major depressive disorder. *Brain Behav. Immun.* **48**, 186–194 (2015).

96. R. R. Stein, V. Bucci, N. C. Toussaint, C. G. Buffie, G. Rätsch, E. G. Pamer, C. Sander, J. B. Xavier, Ecological modeling from time-series inference: Insight into dynamics and stability of intestinal microbiota. *PLoS Comput. Biol.* **9**, e1003388 (2013).

97. J. Mounier, C. Monnet, T. Vallaeys, R. Arditi, A.-S. Sarthou, A. Hélias, F. Irlinger, Microbial interactions within a cheese microbial community. *Appl. Environ. Microbiol.* **74**, 172–181 (2008).

98. D. B. DiGiulio, B. J. Callahan, P. J. McMurdie, E. K. Costello, D. J. Lyell, A. Robaczewska, C. L. Sun, D. S. A. Goltsman, R. J. Wong, G. Shaw, D. K. Stevenson, S. P. Holmes, D. A. Relman, Temporal and spatial variation of the human microbiota during pregnancy. *Proc. Natl. Acad. Sci.* **112**, 11060–11065 (2015).

99. J. Lloyd-Price, C. Arze, A. N. Ananthakrishnan, M. Schirmer, J. Avila-Pacheco, T. W. Poon, E. Andrews, N. J. Ajami, K. S. Bonham, C. J. Brislawn, D. Casero, H. Courtney, A. Gonzalez, T. G. Graeber, A. B. Hall, K. Lake, C. J. Landers, H. Mallick, D. R. Plichta, M. Prasad, G. Rahnavard, J. Sauk, D. Shungin, Y. Vázquez-Baeza, R. A. White III, IBDMDB Investigators, J. Braun, L. A. Denson, J. K. Jansson, R. Knight, S. Kugathasan, D. P. B. McGovern, J. F. Petrosino, T. S. Stappenbeck, H. S. Winter, C. B. Clish, E. A. Franzosa, H. Vlamakis, R. J. Xavier, C. Huttenhower, Multi-omics of the gut microbial ecosystem in inflammatory bowel diseases. *Nature* **569**, 655–662 (2019).

100. F. Turroni, C. Peano, D. A. Pass, E. Foroni, M. Severgnini, M. J. Claesson, C. Kerr, J. Hourihane, D. Murray, F. Fuligni, M. Gueimonde, A. Margolles, G. de Bellis, P. W. O'Toole, D. van Sinderen, J. R. Marchesi, M. Ventura, Diversity of bifidobacteria within the infant gut microbiota. *PLOS ONE* **7**, e36957 (2012).

101. T. Sybille, Z. June, K. Michael, M. Roy, M. L. Marco, The intestinal microbiota in aged mice is modulated by dietary resistant starch and correlated with improvements in host responses. *FEMS Microbiol. Ecol.* **83**, 299–309 (2013).

102. J. Xu, F. Lian, L. Zhao, Y. Zhao, X. Chen, X. Zhang, Y. Guo, C. Zhang, Q. Zhou, Z. Xue, X. Pang, L. Zhao, X. Tong, Structural modulation of gut microbiota during alleviation of type 2 diabetes with a chinese herbal formula. *ISME J.* **9**, 552–562 (2015).

103. A. Rivière, M. Selak, D. Lantin, F. Leroy, L. De Vuyst, Bifidobacteria and butyrate-producing colon bacteria: Importance and strategies for their stimulation in the human gut. *Front. Microbiol.* **7**, 979 (2016).

104. F. De Filippis, N. Pellegrini, L. Vannini, I. B. Jeffery, A. L. Storia, L. Laghi, D. I. Serrazanetti, R. D. Cagno, I. Ferrocino, C. Lazzi, S. Turroni, L. Cocolin, P. Brigidi, E. Neviani, M. Gobbetti, Paul W O'Toole, D. Ercolini, High-level adherence to a mediterranean diet beneficially impacts the gut microbiota and associated metabolome. *Gut* **65**, 1812–1821 (2016).

105. J. Zhang, Z. Guo, Z. Xue, Z. Sun, M. Zhang, L. Wang, G. Wang, F. Wang, J. Xu, H. Cao, H. Xu, Q. Lv, Z. Zhong, Y. Chen, S. Qimuge, B. Menghe, Y. Zheng, L. Zhao, W. Chen, H. Zhang, A phylo-functional core of gut microbiota in healthy young Chinese cohorts across lifestyles, geography and ethnicities. *ISME J.* **9**, 1979–1990 (2015).

106. T. Zhang, Q. Li, L. Cheng, H. Buch, F. Zhang, *Akkermansia muciniphila* is a promising probiotic. *J. Microbial. Biotechnol.* **12**, 1109–1125 (2019).
